# Tracking Familial History of Reading and Math Difficulties in Children’s Academic Outcomes

**DOI:** 10.3389/fpsyg.2021.710380

**Published:** 2022-01-18

**Authors:** Tin Q. Nguyen, Amanda Martinez-Lincoln, Laurie E. Cutting

**Affiliations:** ^1^Vanderbilt Brain Institute, School of Medicine, Vanderbilt University, Nashville, TN, United States; ^2^Department of Special Education, Peabody College of Education and Human Development, Vanderbilt University, Nashville, TN, United States; ^3^Vanderbilt Kennedy Center, Nashville, TN, United States

**Keywords:** familial history, academic, reading, math, intergenerational transmission, reading difficulties, math difficulties

## Abstract

The current study aimed to investigate the extent to which familial history of reading and math difficulties have an impact on children’s academic outcomes within a 3-year longitudinal study, which evaluated their core reading and math skills after first (*N* = 198; 53% girls) and second grades (*N* = 166), as well as performance on complex academic tasks after second and third grades (*N* = 148). At baseline, parents were asked to complete the *Adult Reading History Questionnaire* (ARHQ) and its adaption, *Adult Math History Questionnaire* (AMHQ), to index familial history of reading and math difficulties, respectively. Preliminary findings established the psychometric properties of the AMHQ, suggesting that it is a reliable and valid scale. Correlation analyses indicated that the ARHQ was negatively associated with children’s reading skills, whereas the AMHQ was negatively related to math outcomes. Path results revealed that the ARHQ predicted children’s performance on complex reading tasks indirectly via their core reading skills, and the AMHQ was linked to complex math outcomes indirectly via core math abilities. The ARHQ was also found to be negatively correlated with measures of children’s math performance, with path findings suggesting that these relations were indirectly explained by differences in their core reading skills. These results suggest that assessing familial risk for academic difficulties may be crucial to understanding comorbid etiological and developmental associations between reading and math differences.

## Introduction

Parents’ self-report of academic difficulties, often referred to as familial history, has been shown as a significant predictor of children’s academic outcomes. For example, familial history of reading difficulties has been found to be negatively associated with children’s reading skills and, to some extent, math abilities (Scarborough, 1989; Pennington and Lefly, 2001). However, much remains to be understood regarding the impact of familial math history on academic outcomes, particularly as related to the subcomponents of reading and math, as well as math more generally. As such, the first aim of this study was to build on existing findings by asking whether parents’ self-report of math difficulties, in parallel with familial reading history, negatively predicts differences in children’s academic outcomes. Leveraging a longitudinal design, the second aim was to demarcate the direct and indirect effects of familial history on children’s core reading and math skills versus their performance on complex academic tasks. Results from these research aims could offer diagnostic and intervention implications for children at heightened familial history for academic difficulties, as well as add to the understanding of the comorbid etiological and developmental associations between reading and math differences. (For the list of abbreviations used throughout this study, see Table 1).

**TABLE 1:** List of abbreviations used throughout the current study.

Acronym	Term
**Familial history**	
ARHQ	Adult Reading History Questionnaire
AMHQ	Adult Math History Questionnaire
**Core academic skills**	
WR	Word recognition
AR	Arithmetic calculation
**Complex academic tasks**	
RC	Reading comprehension
PS	Problem solving

### Parents’ Self-Report of Academic Difficulties

One way to capture familial history of academic difficulties is by parents’ self-report, which has been shown to be reliable (e.g., Lefly and Pennington, 2000). Some studies have utilized a dichotomous, or yes-versus-no, indicator for familial history of reading and/or math difficulties (Landerl and Moll, 2010; Erbeli et al., 2019; Khanolainen et al., 2020). Parents’ self-report of academic difficulties, operationalized as a dichotomous variable, has indeed been shown to negatively predict children’s reading and math outcomes. These findings have revealed that children with parents who self-report childhood difficulties when learning to read words or performing arithmetic computation in elementary school as compared to children whose parents report no such difficulties are more likely to exhibit differences in their academic skills when they start their formal education (e.g., Landerl and Moll, 2010; Erbeli et al., 2019; Khanolainen et al., 2020). Nevertheless, the strategy in treating familial history of academic difficulties as a dichotomous variable has been caveated to be somewhat arbitrary because “the liability distribution for a given disease is often continuous and quantitative” (Snowling et al., 2003; Pennington, 2006). To this end, some studies have used more in-depth questionnaires, such as the *Adult Reading History Questionnaire* (ARHQ), to refine the specificity, sensitivity, as well as severity in terms of reading-related differences in a dimensional manner (Lefly and Pennington, 2000; see also Welcome and Meza, 2019). The ARHQ, a revision of an earlier self-report designed by Finucci et al. (1982), includes items that query not only childhood reading difficulties, but also previous school experiences, attitude toward reading, as well as current literacy practices among adult responders or parents (Lefly and Pennington, 2000). Findings reveal substantial correlations among these items within the ARHQ (Lefly and Pennington, 2000; Welcome and Meza, 2019), thus supporting the approach of assessing familial history, at least within the reading domain, in a dimensional and continuous manner. For example, studies have reported that higher scores on the ARHQ are prospectively associated with worse performance across reading and, although evidence for this is limited, even math tasks (Pennington and Lefly, 2001). Such predictive effects of the ARHQ has further been implicated to be independent from some children’s eventual status of reading disability (dyslexia) and parents’ level of educational attainment (Pennington and Lefly, 2001), and have been replicated in a range of studies and designs (neurobiological: Black et al., 2012; twin: Rosenberg et al., 2012; and genotyping: Stefansson et al., 2014), motivating the utility of this scale in underscoring the continuous nature and predictive effect of familial reading history in reading and perhaps math outcomes.

While the ARHQ has fairly robust empirical support at this time, findings with regard to familial history of math difficulties measured in a dimensional, continuous manner have not yet been reported, as there is currently no validated scale that captures familial history of math difficulties that mirrors the ARHQ^1^. To address this gap in the literature, the *Adult Math History Questionnaire* was designed and implemented when the current longitudinal study commenced in 2015 in order to track the role of familial math history in children’s academic outcomes over time.

### Familial History of Reading Difficulties

The predictive effect of familial reading history has been characterized in terms of children’s emergent literacy processes and later reading outcomes. Evidence has revealed that presence of familial reading difficulties is negatively associated with children’s letter-word knowledge and phonological awareness (Pennington and Lefly, 2001; Carroll and Snowling, 2004; Giménez et al., 2017). These are important emergent literacy processes that provide children with the linguistic foundation prior to formal education and prepare for when they learn how to read (Storch and Whitehurst, 2002). Familial history of reading difficulties has indeed been shown to negatively predict word recognition (WR) differences in children starting elementary school – i.e., where and when they receive explicit reading instruction (Ehri, 2005; Common Core State Standards Initiative, 2010a). Furthermore, results from path analyses have highlighted the indirect impact of familial reading history on children’s WR skills through their emergent literacy processes (Solari et al., 2018; Esmaeeli et al., 2019). These reports are consistent with various reading frameworks, including the core versus multiple deficit hypotheses (Pennington, 2006; Melby-Lervåg et al., 2012; van Bergen et al., 2014) that speculate for the distinguishable effects of familial history on developmental predictors and subskills in reading.

Later in school, children’s WR proficiency is a critical predictor of their performance on complex academic tasks such as reading comprehension (RC) (García and Cain, 2014); given that RC is key to children’s future educational and vocational outcomes (Ritchie and Bates, 2013), understanding the extent to which familial history of reading difficulties over the course of development plays a role in the endpoint of reading – RC – is of substantial importance. While some studies have shown that familial history of reading difficulties negatively predicts children’s performance on RC tasks (Pennington and Lefly, 2001; van Viersen et al., 2018), such predictive effect of familial history in children’s RC outcomes appeared to be substantially reduced when measures of their core reading abilities, including WR, are included (Conlon et al., 2006; Solari et al., 2018). Results from longitudinal path analyses have indeed shown that familial history of reading difficulties relates to children’s RC performance indirectly via their WR skills (Hulme et al., 2015). Much less is known about the influence of familial reading history on math outcomes, and most centrally whether familial history of math difficulties is linked to children’s math performance. Such an approach that distinguishes differential effects on various academic domains (reading versus math) as well as levels of academic outcomes (core versus complex; Cirino et al., 2018; Child et al., 2019) could highlight the extent to which aspects of familial history of specific academic difficulties explains the etiological differences in children’s learning and cognitive profiles.

### Familial History of Math Difficulties

Some evidence, although much more limited than in the reading domain, has implicated a link between familial history of math difficulties and children’s math outcomes. Just as complex reading tasks are known to rely on children’s WR proficiency, a core skill to math performance is arithmetic calculation (AR), or the ability to solve single- and often multi-digit addition and subtraction tasks (Fuchs et al., 2006; Cirino et al., 2018). Similar to WR, children are introduced to AR and the procedural aspect of math performance in the first years of formal education (Common Core State Standards Initiative, 2010b). Studies using a performance-based operationalization of math difficulties from family members have observed comparable differences in AR skills between children with math difficulties (dyscalculia) and their family members, i.e., parents and siblings (Shalev et al., 2001). While not with self-report, these findings nonetheless suggest initial yet compelling evidence for a role of familial history in children’s math outcomes (Shalev et al., 2001; Geary, 2011). In a recent study, a dichotomous measure of parents’ self-report of math, but not reading, difficulties was demonstrated to negatively predict children’s performance on timed AR tasks (Khanolainen et al., 2020). As they advance in school, children continue to build on their AR proficiency when working on complex math tasks, namely problem solving (PS), which generally includes linguistically presented arithmetic prompts (Fuchs et al., 2006; Common Core State Standards Initiative, 2010b). However, insufficient data are available to determine whether familial history of math difficulties might play a role in children’s performance on PS tasks.

In sum, scarce but compelling evidence prompts a need for the *Adult Math History Questionnaire* (AMHQ). The AMHQ would be expected to capture the continuous nature as well as predictive effect of familial history of math difficulties. By referencing and adapting the ARHQ (Lefly and Pennington, 2000), items in the AMHQ were designed to tap childhood math difficulties, school experiences with math-related materials, attitude toward math, and current numeracy practices. Specifically, *Items 1*, *2*, *3*, and *4* survey the respondents’ experiences with math learning and related contents in elementary school, whereas *Items 5* and *6* with materials in post-primary education (high school and college). *Items 7*, *8*, and *10* target the respondents’ (current) attitude toward math and related contents, while *Items 14*, *15*, *16*, and *17* also place an emphasis on confidence and interest in math. *Items 9*, *11*, *12*, and *13* inquire about the respondents’ current numeracy practices and math exposure. Using the ARHQ and AMHQ in parallel enables mapping the overlapping versus unique impacts that familial history of reading versus math difficulties might have on children’s academic outcomes. This is critical, as previous studies suggest an indirect predictive effect via WR skills of the ARHQ on children’s RC performance. Therefore, it is plausible that familial history of math difficulties, indexed by the AMHQ in this study, could have an indirect association with children’s PS performance via AR skills. Moreover, in view of the multiple deficit hypothesis (Pennington, 2006; van Bergen et al., 2014), along with prior findings on the impact of familial history of reading difficulties on children’s math performance (Pennington and Lefly, 2001), the ARHQ and AMHQ could have cross-domain effects.

### Comorbid Reading and Math Differences

Difficulties in reading and math co-occur more often than differences in either domain alone (e.g., Dirks et al., 2008; Landerl and Moll, 2010); yet, the etiological basis of their comorbid association, especially in terms of familial history, remains unclear. As aforementioned, studies have observed the negative associations between scores on the ARHQ and both reading and math outcomes in children (Pennington and Lefly, 2001). Other studies that have tracked parents’ self-report of math difficulties, though as a dichotomous indicator, have found differences in children’s performance on math and, to a lesser extent, on reading (Landerl and Moll, 2010). These findings are consistent with previous suggestions that difficulties in one academic domain could exacerbate concerns in another (Jordan, 2007). It bears noting that while the rates of comorbid academic differences differ between population-based twin studies, what is fairly consistent is the percentage of children with math difficulties showing reading challenges is relatively higher than that of those with reading problems exhibiting math struggles (e.g., Dirks et al., 2008; Landerl and Moll, 2010). This may be because children with familial history of reading difficulties may not adequately meet the verbal demands in math tasks (Amland et al., 2021; also see Moll et al., 2015).

Differentiating various levels of academic outcomes, as previously remarked, could be crucial to understanding the etiological differences in children’s learning and cognitive profiles. Comorbid differences in core academic skills, i.e., WR and AR, have been thought by some to stem from familial transmission of procedural learning difficulties (Light and DeFries, 1995; Niemi et al., 2011). With respect to the reported rates of comorbidity in math versus reading outcomes, it could be that differences in WR abilities mediate the relation between familial history of academic difficulties and AR skills (see Pennington and Lefly, 2001; Landerl and Moll, 2010; Moll et al., 2015). Studies have observed some genetic overlapping between children’s WR abilities and their performance on PS tasks (Hart et al., 2009). RC and PS outcomes are also substantially associated (Compton et al., 2012; Fuchs et al., 2018). Emerging evidence asserts that RC performance is a better predictor of PS outcome, than vice versa, largely due to both verbal (linguistic) and non-verbal (reasoning) demands in applied math tasks (Fuchs et al., 2018; Spencer et al., 2020). What remains elusive is the knowledge about whether and, if so, to what extent familial history of academic difficulties contributes to the comorbid differences in children’s performance on complex tasks. Based on comorbidity and prediction findings, we hypothesized that familial history of reading difficulties would have an indirect impact on children’s math outcomes through their reading abilities.

### Current Study and Specific Aims

Broadly, the present study focused on understanding the extent to which familial history of academic difficulties have an impact on children’s reading and math outcomes by using data from a 3-year longitudinal study that collected parental self-report of academic difficulties at baseline and assessed children’s core academic skills (WR and AR) after first and second grades, as well as performance on complex academic tasks (RC and PS) after second and third grades. The first aim was to replicate and extend previous findings by examining the relations between parents’ self-reports of academic difficulties (ARHQ and AMHQ) and children’s reading and math outcomes, including establishing psychometric properties of the AMHQ. With correlation analyses, scores on the ARHQ were expected to be associated with children’s reading abilities, whereas the AMHQ were hypothesized to be linked to their math skills.

The second aim examined the extent to which familial history of reading versus math difficulties would predict differences in children’s performance on complex tasks directly or indirectly via their core skills (see Supplementary Figure 1). Path analyses were used in order to take into account the developmental associations within and between academic skills, i.e., their autoregressions and covariances, respectively (Erbeli et al., 2019). At the same time, the current longitudinal design allowed for evaluating the cross-lagged effects that core academic skills (collected after first and second grades) would predict performance on complex tasks (evaluated after second and third grades). The ARHQ was hypothesized to indirectly predict children’s RC performance via differences in WR skills, while the AMHQ was hypothesized to predict PS outcomes via AR abilities. Particular attention was paid toward observing whether there might be overlapping versus unique impacts from familial history of reading versus math difficulties on children’s reading and/or math outcomes, with the hypothesis that familial history of reading difficulties would impact math skills; however, we were agnostic as to whether the same cross-domain effects would be present for familial history of math difficulties.

## Materials and Methods

### Participants and Procedure Overview

The current study and related procedures were carried out in accordance with the Institutional Review Board at (DBPR). Participants were recruited from local schools, clinics, and pediatrician’s officers as well as the greater (DBPR). All participants were native English speakers, with normal or correctable visual or auditory differences, and did not demonstrate history or presence of a pervasive development disorder or known neurological disorder. Participants with ADHD were not excluded, provided that they could sustain attention for assessments. Upon enrollment, children provided informed assent, and their parents completed written consent. [Additional information on this longitudinal sample can be found in (DBPR); (DBPR)].

Data were drawn from *N* = 198 children after their successful completion of first grade (*m*_age_ = 7.47, *sd* = 0.36, *range* = 6.42–8.33). 105 (53%) were girls. 5 (3%) were Asian, 23 (12%) Black, 150 (76%) White, 16 (8%) more than one race, and 4 (2%) reported as others. 10 (5%) reported as Hispanic/Latino. Information about the school that children attended was collected by identifying whether or not it receives Title 1 Federal Supplement (i.e., with more than 40% of students receiving free or reduced-price lunch, living below the poverty line) to accommodate educational activities, based on publicly available data [(DBPR); as done in, e.g., Del Tufo et al., 2019]. *N* = 166 (84% of 198) children returned after second grade, and *N* = 148 (89% of 166) after third grade, with approximately a year between visits. Children’s IQ was measured once at baseline, using both Vocabulary and Matrix Reasoning subtests from the *Wechsler Abbreviated Scale for Intelligence* (Wechsler, 2011). Descriptive information for the current longitudinal sample can be found in Table 2.

**TABLE 2:** Descriptive statistics for the current longitudinal sample, including information on parental measures [reading history (ARHQ), math history (AMHQ), and educational attainment] and children’s demographic variables [age, IQ (at baseline), sex, and school information (Title 1 Status)], core academic skills [word recognition (WR) and arithmetic calculation (AR)], and performance on complex tasks [reading comprehension (RC) and problem solving (PS)].

		*M*	*Sd*	*Min*	*Max*
**Parental measures**					
(1)	Reading history (ARHQ)	27.78	13.48	3	74
(2)	Math history (AMHQ)	31.04	18.57	0	80
(3)	Educational attainment	6.10	0.88	3	7
**Child measures**					
**Demographic variables**					
(4)	Age (after 1st grade)	7.47	0.36	6.42	8.33
(5)	IQ	104.66	13.82	60	136
(6)	Sex	105 (53%) girls			
(7)	School (Title 1 Status)	35 (17%) attended			
**Core academic skills**					
(8)	WR (after 1st grade)	477.39	19.42	413	519
(9)	WR (after 2nd grade)	490.99	16.21	443	530
(10)	AR (after 1st grade)	452.34	12.60	401	486
(11)	AR (after 2nd grade)	466.70	14.11	427	506
**Complex academic tasks**					
(12)	RC (after 2nd grade)	484.50	13.26	443	515
(13)	RC (after 3rd grade)	494.68	13.05	460	521
(14)	PS (after 2nd grade)	493.04	17.46	431	531
(15)	PS (after 3rd grade)	503.86	34.93	116	534

*Data were drawn from N = 198 children after first grade, N = 166 after second, and N = 148 after third. [W scores from Woodcock et al. (2001, 2007) on child measures were used].*

### Parental Measures

Self-report data on familial history of reading and math difficulties as well as educational attainment were collected from parents once using questionnaires at the first visit (i.e., when children were enrolled in the study after first grade). Additionally, performance-based measures of academic skills were administered to parents in order to establish the psychometric properties of their self-report data in supplemental analyses.

#### Familial History

For reading history, parents were asked to complete the *Adult Reading History Questionnaire* (ARHQ; Lefly and Pennington, 2000). The ARHQ contained 23 items (see Supplementary Table 1), where each used a five-point Likert scale and higher score would indicate increased likelihood of familial history of reading difficulties. For example, for (*Item 2*) “How much difficulty did you have learning to read in elementary school?”, the responses would range from 0 = “None” to 4 = “A great deal.” Partial credit was acknowledged with 0.5-point increment.

For math history, parents were asked to complete the *Adult Math History Questionnaire* (AMHQ), which was adapted from the ARHQ (Lefly and Pennington, 2000; see also Stefansson et al., 2014; Ulfarsson et al., 2017). The AMHQ contained 17 five-point items, with partial credit of 0.5-point increment (see Supplementary Table 2), where higher score would indicate increased likelihood of math difficulties. For example, for (*Item 4*) “Compared to others in your elementary classes, how much did you struggle to complete your math work?”, the responses would range from 0 = “Not at all” to 4 = “Much more than most.” (See also “Supplementary Materials and Methods” for the descriptive information on individual items from the AMHQ, what they were purported to capture, and how they might overlap with or differ from another scale of this kind).

#### Academic Skills

For reading, the Letter-Word Identification, Word Attack, and Sentence Reading Fluency from the *Woodcock-Johnson III* (WJ-III; Woodcock et al., 2001, 2007) were administered to measure parents’ ability to identify isolated real words and apply phonic skills to decode non-words (untimed), as well as read and comprehend simple sentences (timed), respectively, all of which were used to calculate the composite score (Basic Reading cluster from the WJ-III).

For math, the Calculation and Math Facts Fluency subtests also from the WJ-III (Woodcock et al., 2001, 2007) were administered to estimate parents’ ability to perform basic mathematical operations (untimed) and apply calculation skills to single-digit numbers (timed), both of which were used to compute the composite score (Math Calculation cluster from the WJ-III).

#### Educational Attainment

Parents were asked to report their highest level of educational attainment, which was then rated on a seven-point scale, where 1 = “*less than seventh grade*,” 2 = “*junior high school (ninth grade),”* 3 = “*partial high school (tenth or eleventh grade*),” 4 = “*high school graduate (whether private preparatory, parochial, trade, or public school)*,” 5 = “*partial college (at least 1 year) or specialized training*,” 6 = “*standard college or university graduation*,” or 7 = “*graduate professional training (graduate degree).*”

### Child Measures

Performance data on core academic skills (word recognition and arithmetic calculation) were acquired from children after first and second grades using standardized measures, whereas complex academic tasks (text comprehension and word-problem solving) were administered after second and third grades. *W* scores from child measures were used in analyses. A *W* score is purported to represent both person-level ability and item-level difficulty on the same equal-interval scale and thought to be suitable for longitudinal modeling strategies (Woodcock et al., 2001, 2007).

#### Core Academic Skills

For word recognition (WR), the Letter-Word Identification and Word Attack subtests from the WJ-III (Woodcock et al., 2001, 2007) were administered to assess children’s ability to recognize real words and decode non-words, respectively, both of which were used to calculate the composite score (Basic Reading cluster from the WJ-III) for analyses.

For arithmetic calculation (AR), the Calculation subtest also from the WJ-III (Woodcock et al., 2001, 2007) was administered to evaluate children’s number knowledge and ability to perform basic algebraic computation.

#### Complex Academic Tasks

For reading comprehension (RC), the Passage Comprehension subtest from the WJ-III (Woodcock et al., 2001, 2007) was administrated to measure children’s ability to read, relate ideas, and fill in missing words (modified cloze).

For problem solving (PS), the Applied Problems subtest from the WJ-III (Woodcock et al., 2001, 2007) was administered to capture children’s quantitative reasoning and ability to solve orally presented problems.

### Statistical Strategies

Analyses were performed in *R* (with publicly available packages indicated where appropriate).

#### Psychometric Analyses

Self-report data from parents (on the ARHQ and AMHQ, separately) were subjected to three sets of preliminary analyses to (1) establish the reliability of each scale as a whole and at the level of individual items, (2) explore the factor structure of each scale, and (3) evaluate the correlations between scores (total and factor) on each scale and performance-based measures of academic skills. Analyses were conducted using the *psych* and *scale* packages (Revelle, 2018).

• First, for reliability analyses, after reporting their descriptive statistics, questionnaire items were individually correlated with the total score (i.e., item-total correlation) and corrected for scale reliability. Each item was reported with item-rest correlation and Cronbach’s α if it were to be dropped. Pairwise correlations were examined among items within and between the ARHQ and AMHQ, along with their corresponding total scores.
• Second, to explore their factor structure, the ARHQ and AMHQ were each analyzed following steps previously taken in Welcome and Meza (2019; particularly for the ARHQ and the naming convention for its derived factors), which include: establishing the *KMO* (Kaiser–Meyer–Olkin) value of sampling adequacy, conducting the *BTS* (Bartlett’s Test of Sphericity) for suitability in capturing sample variance, visualizing scree plot to estimate the number of factors to extract, and performing maximum likelihood method with oblique rotation (direct oblimin) to derive the respective components, which were rendered through a regression-based approach to calculate factor scores (Tabachnick and Fidell, 2007; DiStefano et al., 2009). Note that the directionality for the computed factor scores remain consistent with the directionality of the questions on each scale – that is, higher scores on any factors extracted from the ARHQ or AMHQ indicate, e.g., increased difficulties with learning to read or do simple arithmetic.
• Third, total scores on the ARHQ and AMHQ, as well as their factor scores to be derived from these scales, were subjected to correlation analyses with performance-based measures of parents’ reading and math skills as supplemental findings. Total scores on the ARHQ and AMHQ were used in the following formal correlation and path analyses to index familial history of reading and math difficulties, respectively.

#### Correlational Analyses

Analyses were conducted to evaluate the pairwise associations among parental measures [reading history (ARHQ), math history (AMHQ), and educational attainment] and children’s demographic variables [age, IQ (at baseline), sex, and school information (Title 1 Status)], core academic skills (WR and AR), and performance on complex tasks (RC and PS) across visits. At the same time, results from these correlational analyses would reveal the validity of the ARHQ and AMHQ in relation to performance-based measures of children’s academic performance. Supplementary analyses were also performed to assess the correlations between scores on individual factors derived from the ARHQ and AMHQ and measures of children’s academic performance.

#### Path Analyses

Path models were constructed to determine the direct and indirect effects of familial history of reading and math difficulties on children’s core academic skills and performance on complex academic tasks. Analyses were conducted in two ways: first, using the total scores on the ARHQ and the AMHQ; and second, with scores for individual factors to be derived from these scales. Consistent with previous literature, using total scores on the ARHQ to analyze with children’s academic outcomes captures the continuous nature of familial reading history over time and across contexts (Lefly and Pennington, 2000; Welcome and Meza, 2019). Similar to this line of reasoning, findings from analyzing total scores on the AMHQ and children’s academic outcomes would also illustrate the continuous nature of familial math history based on parents’ self-reported experiences over time and across contexts that involve general math learning and numeracy practices. Subsequently, analyses with factor scores derived from the ARHQ and AMHQ then enable a more granular understanding of the impact of familial reading and/or math history by differentiating which specific components, such as difficulties with learning in childhood or current literacy/numeracy practices, could have driven the overall associations between familial reading and/or math history and children’s academic outcomes.

Variables included in correlational analyses were submitted to path modeling using the *lavaan* package (Rosseel, 2012). Familial history of reading and math difficulties were directly mapped onto children’s core academic skills measured after first and second grades, as well as onto their academic performance assessed after second and third grades. All variables were adjusted for parents’ educational attainment and children’s demographic variables [age, IQ (at baseline), sex, and school information (Title 1 Status)]. Then, longitudinal paths were represented for measures of children’s academic profile across the three visits, where core skills were treated as the longitudinal mediators for the indirect effects of familial history of academic difficulties on complex tasks. Covariances were specified for pairs of predictors between reading and math domains – i.e., between familial reading and math history (ARHQ and AMHQ), between WR and AR, and between RC and PS. Finally, any non-significant paths were constrained to zero to yield the final model. Then, the standard errors, and thus levels of significance, were inferred using the bootstrapping approach (Fritz et al., 2012; Rosseel, 2012). For each model, fit was determined by non-significant χ^2^ (chi-square), *CFI* and *TLI* (Comparative Fit and Tucker–Lewis Indices) greater than or equal to 0.95, and *RSMEA* (Root Mean Square Error of Approximation) and *SRMR* (Standardized Root Mean Square Residual) values less than 0.05 (Hu and Bentler, 1999). Supplementary analyses were also conducted by repeating the outlined path modeling strategies to model the effects of individual factors derived from the ARHQ and AMHQ and measures of children’s academic performance.

## Results

Descriptive statistics on the current longitudinal sample can be found in Table 2, which includes information on parental measures [reading history (ARHQ), math history (AMHQ), and educational attainment] and children’s demographic variables [age, IQ (at baseline), sex, and school information (Title 1 Status)], core academic skills [word recognition (WR) and arithmetic calculation (AR)], and performance on complex tasks [reading comprehension (RC) and problem solving (PS)]. [Additional findings (from intermediate steps or follow-up analyses) are available for viewing in conjunction with this Section “Results” and can be found in the Supplementary Results].

### Psychometric Findings

Reliability analyses were conducted on self-report data from parents on the ARHQ and AMHQ. Since there is not yet a scale capturing familial history of math difficulties, the AMHQ was adapted from the ARHQ (Lefly and Pennington, 2000) with the intention that the AMHQ would translate items in the ARHQ to estimate math- rather than reading-related contents. To this end, analyses on the AMHQ were conducted in parallel with data from the ARHQ to attest to their reliability as well as validity properties.

#### Adult Reading History Questionnaire

• The ARHQ was reported with Cronbach’s α = 0.87, with 95% confidence interval of (0.85, 0.90), suggesting good internal consistency. Descriptive and reliability statistics on individual items can be found in Supplementary Table 1. Briefly, (*Item 2*) “How much difficulty did you have learning to read in elementary school?” and (*Item 6*) “How would you compare your reading skill to that of others in your elementary classes?” appeared to demonstrate the highest correlations with the total score on the ARHQ (item-total *r*’s = 0.727 and 0.701, respectively, after corrected for scale reliability). In contrast, (*Item 23*) “Do you read a newspaper on Sunday?” and (*Item 21*) “Do you read daily (Monday–Friday) newspapers?” appeared to have the lowest correlations with the total score (item-total *r*’s = 0.246 and 0.217, after corrected for scale reliability). Pairwise correlations among items in the ARHQ are reported in Supplementary Table 3 and with those from the AMHQ in Supplementary Table 4.
• The KMO coefficient was reported with 0.81, and the BTS was significant (χ^2^ = 2275.697, *p* < 0.01), indicating adequate sampling as well as suitability to capture the sample’s variability. The scree plot suggested a six-factor solution, wherein the oblique rotation yielded the following: (*Factor 1*) Childhood Ability, (*Factor 2*) Attitude/Exposure, (*Factor 3*) Memory, (*Factor 4*) Media Use, (*Factor 5*) Reversal, and (*Factor 6*) Spelling. [Findings from Welcome and Meza (2019) were used to guide the naming convention for factors derived in the current study]. Loading coefficients from individual items for these factors can be found in Table 3 and Supplementary Results.

**TABLE 3:** Factor loadings for individual items in the structure revealed from the ARHQ, wherein the solution was reported with (*Factor 1*) Childhood Ability, (*Factor 2*) Attitude and Exposure, (*Factor 3*) Memory, (*Factor 4*) Media Use, (*Factor 5*) Reversal, and (*Factor 6*) Spelling.

Item	Description	Childhood ability	Attitude and exposure	Memory	Media Use	Reversal	Spelling
**Adult Reading History Questionnaire**							
1	Which of the following most nearly describes your attitude toward school when you were a child?	0.398	0.118	0.156	–	−0.133	–
2	How much difficulty did you have learning to read in elementary school?	0.822	–	–	–	–	–
3	How much extra help did you need when learning to read in elementary school?	0.832	–	–	–	–	–
4	Did you ever reverse the order of letters or numbers when you were a child?	–	–	–	–	0.995	–
5	Did you have difficulty learning letter and/or color names when you were a child?	0.518	–	–	–	0.382	−0.146
6	How would you compare your reading skill to that of others in your elementary classes?	0.697	–	–	–	–	–
7	All students struggle from time to time in school. In comparison to others in your classes, how much did you struggle to complete your work?	0.638	–	0.173	–	–	0.116
8	Did you experience difficulty in high school or college English classes?	0.553	0.126	–	–	–	–
9	What is your current attitude toward reading?	0.209	0.561	–	–	–	–
10	How much reading do you do for pleasure?	–	0.952	–	–	–	–
11	How would you compare your current reading speed to that of others of the same age and education?	0.236	0.238	–	0.135	–	0.220
12	How much reading do you do in conjunction with your work? (If retired or not working, how much did you read when you were working?)	–	0.159	0.135	0.215	–	0.183
13	How much difficulty did you have learning to spell in elementary school?	0.249	–	0.108	–	0.189	0.591
14	How would you compare your current spelling to that of others of the same age and education?	–	–	–	–	–	0.913
15	Did your parents ever consider having you repeat any grades in school due to academic failure (not illness)?	0.718	–	–	–	−0.157	–
16	Do you ever have difficulty remembering people’s names or names of places?	–	–	0.901	–	–	–
17	Do you ever have difficulty remembering addresses, phone numbers, or dates?	–	–	0.717	–	–	–
18	Do you have difficulty remembering complex verbal instructions?	0.131	–	0.649	–	–	–
19	Do you currently reverse the other of letters or numbers when you read or write?	0.120	–	0.122	–	0.491	–
20	How many books do you read for pleasure each year?	–	0.849	–	–	–	–
21	How many magazines do you read for pleasure each month?	–	–	–	0.456	–	0.117
22	Do you read daily (Monday–Friday) newspapers?	–	–	–	0.725	–	0.117
23	Do you read a newspaper on Sunday?	–	–	–	0.918	–	–

	SS loadings	3.723	2.095	1.854	1.681	1.496	1.375
	Proportion variance	0.162	0.091	0.081	0.073	0.065	0.060
	Cumulative variance	0.162	0.253	0.334	0.407	0.472	0.531

*[Findings from Welcome and Meza (2019) were used to guide the naming convention for factors derived in the current study].*

• Analyses with performance-based measures of parents’ academic skills revealed that scores on the ARHQ were significantly and negatively correlated with reading scores (*r*’s = −0.31 – −0.43, *p* < 0.05) (see Supplementary Table 7). Additionally, scores on the ARHQ were significantly and negatively correlated with math scores (*r*’s = −0.20 – −0.30, *p* < 0.05). Detailed discussion on the correlations between factor scores derived from the ARHQ and performance-based measures of parents’ academic skills can be found in Supplementary Results. Briefly, the Childhood Ability, Attitude/Exposure, Reversal, and Spelling factors derived from the ARHQ were associated with parents’ reading skills, while the Childhood Ability, Reversal, and Spelling ones were correlated with their reading and math abilities.

#### Adult Math History Questionnaire

• The AMHQ was reported with Cronbach’s α = 0.93, with 95% confidence interval of (0.91, 0.94), suggesting excellent internal consistency. Descriptive and reliability statistics on individual items from the AMHQ in Supplementary Table 2. Briefly, (*Item 8*) “Math makes me feel uncomfortable and nervous.” and (*Item 4*) “Compared to others in your elementary classes, how much did you struggle to complete your math work?” had the highest correlations with the total score on the AMHQ (item-total *r*’s = 0.852 and 0.803, respectively, after corrected for scale reliability). In contrast, (*Item 11*) “My current work requires I use math.” and (*Item 15*) “I would like to further develop my math skills.” were reported with the lowest correlations with the total score (item-total *r*’s = 0.422 and 0.393, after corrected for scale reliability). Pairwise correlations among items in the AMHQ are reported in Supplementary Table 5 and with those from the ARHQ in Supplementary Table 6.
• In the initial steps, the KMO coefficient was reported with 0.91, and the BTS was significant (χ^2^ = 2333.652, *p* < 0.01). The scree plot suggested a three-factor solution. Though, findings from the oblique rotation revealed that *Item 13* loaded poorly or out of range (i.e., absolute value > 1.000), thus prompting the decision to omit this item in subsequent steps for factor analyses. After omitting *Item 13*, the KMO coefficient was reported with 0.93, and the BTS was significant (χ^2^ = 2165.691, *p* < 0.01). The scree plot suggested a two-factor solution, wherein the oblique rotation yielded the following: (*Factor 1*) Attitude/Exposure and (*Factor 2*) Childhood Ability. Loading coefficients from individual items for these factors can be found in Table 4 and Supplementary Results.

**TABLE 4:** Factor loadings for individual items in the structure revealed from the AMHQ, wherein the solution was reported with (*Factor 1*) attitude and exposure and (*Factor 2*) childhood ability.

Item	Description	Attitude and exposure	Childhood ability
**Adult Math History Questionnaire**			
1	When in elementary school, I struggled with learning new concepts in math.	–	0.914
2	When in elementary school, I needed extra help in math from a teacher or tutor.	−0.113	0.965
3	How would you compare your math skills to those of others in your elementary classes?	0.190	0.678
4	Compared to others in your elementary classes, how much did you struggle to complete your math work?	0.151	0.803
5	During high school or college, I struggled in math courses.	0.508	0.385
6	I took math classes in high school or college that were not required because I enjoyed them.	0.544	–
7	What is your current attitude toward math?	0.896	−0.114
8	Math makes me feel uncomfortable and nervous.	0.814	0.116
9	As an adult, I struggle to complete math-related tasks, such as calculating tips.	0.499	0.281
10	Math is important in everyday life.	0.402	–
11	My current work requires I use math.	0.456	−0.104
12	I enjoy completing math and logic puzzles for fun.	0.736	–
13	I use math in my everyday life.	(Omitted)	(Omitted)
14	How would you compare your current math skills compared to those of others of the same age and education?	0.755	–
15	I would like to further develop my math skills.	0.523	−0.174
16	I feel confident in helping my child with their math and homework.	0.667	–
17	New math content has usually been easy and enjoyable for me to understand.	0.709	0.163

	SS loadings	5.042	3.205
	Proportion variance	0.315	0.200
	Cumulative variance	0.315	0.515

• Analyses with performance-based measures of parents’ academic skills revealed that scores on the AMHQ were significantly and negatively correlated with math scores (*r*’s = −0.41 – −0.53, *p* < 0.05) (see Supplementary Table 7). There was a weak but significant and negative correlation between scores on the AMHQ and some reading scores (*r*’s = −0.17, *p* < 0.05). Discussion regarding the correlations between factor scores derived from the AMHQ and performance-based measures of parents’ academic skills can be found in Supplementary Results. Briefly, the Attitude/Exposure factor derived from the AMHQ was only associated with parents’ math abilities, whereas the Childhood Ability one was correlated with both their math and reading skills.

### Correlational Findings

Pairwise correlations among parental measures [reading history (ARHQ), math history (AMHQ), and educational attainment] and children’s demographic variables [age, IQ (at baseline), sex, and school information (Title 1 Status)], core academic skills (WR and AR), and performance on complex tasks (RC and PS) can be found in Table 5. Total scores on the ARHQ and AMHQ (reading history versus math history, respectively) were significantly and positively correlated (*r* = 0.33, *p* < 0.05), implicating an association between familial history of reading and math difficulties, respectively. Pairwise correlations between scores on individual factors derived from the ARHQ and AMHQ and measures of children’s academic performance can be found in Supplementary Table 8 and are discussed in Supplementary Results. As noted in Section “Materials and Methods,” when interpreting findings that pertain to the computed factor scores, their directionality remain consistent with the that of the questions on each scale – that is, higher scores on any factors extracted from the ARHQ or AMHQ indicate, e.g., increased difficulties with learning to read or do simple arithmetic.

**TABLE 5:** Pairwise correlations among parental measures [reading history (ARHQ), math history (AMHQ), and educational attainment] and children’s demographic variables [age, IQ (at baseline), sex, and school information (Title 1 Status)], core academic skills [word recognition (WR) and arithmetic calculation (AR)], and performance on complex tasks [reading comprehension (RC) and problem solving (PS)].

		1	2	3	4	5	6	7	8	9	10	11	12	13	14	15
**Parental measures**																
(1)	Reading history (ARHQ)	–														
(2)	Math history (AMHQ)	**0.33**	–													
(3)	Educational attainment	**−0.23**	**−0.21**	–												
**Child measures**																
**Demographic variables**																
(4)	Age (after 1st grade)	0.01	**0.15**	−0.04	–											
(5)	IQ	**−0.20**	−0.02	**0.28**	**−0.23**	–										
(6)	Sex	−0.08	−0.01	0.06	0.09	−0.12	–									
(7)	School (title 1 status)	**0.15**	**0.24**	**−0.28**	−0.02	**−0.23**	0.04	–								
**Core academic skills**																
(8)	WR (after 1st grade)	**−0.19**	−0.08	**0.22**	0.06	**0.43**	0.00	**−0.21**	–							
(9)	WR (after 2nd grade)	**−0.17**	−0.04	**0.25**	0.02	**0.39**	0.05	**−0.25**	**0.89**	–						
(10)	AR (after 1st grade)	**−0.14**	**−0.17**	**0.25**	0.07	**0.41**	0.06	**−0.17**	**0.57**	**0.50**	–					
(11)	AR (after 2nd grade)	**−0.20**	**−0.29**	**0.31**	**0.16**	**0.30**	0.10	−0.15	**0.46**	**0.42**	**0.72**	–				
**Complex academic tasks**																
(12)	RC (after 2nd grade)	**−0.18**	0.00	**0.23**	0.01	**0.46**	−0.03	**−0.17**	**0.68**	**0.71**	**0.46**	**0.37**	–			
(13)	RC (after 3rd grade)	**−0.17**	−0.06	**0.23**	0.00	**0.50**	0.01	**−0.27**	**0.57**	**0.61**	**0.37**	**0.38**	**0.77**	–		
(14)	PS (after 2nd grade)	**−0.26**	−0.13	**0.39**	0.13	**0.53**	0.09	**−0.27**	**0.57**	**0.54**	**0.60**	**0.61**	**0.62**	**0.56**	–	
(15)	PS (after 3rd grade)	−0.13	0.00	0.14	−0.09	**0.17**	−0.04	−0.06	0.12	0.15	**0.28**	**0.25**	**0.19**	**0.55**	**0.32**	–

*(Correlation coefficients in bold met p < 0.05).*

#### Familial History of Reading Difficulties

##### Total Scores

Familial history of reading difficulties, as indexed by total scores on the ARHQ, was significantly correlated negatively with parents’ educational attainment (*r* = −0.23), and negatively with children’s IQ (*r* = −0.20) and positively with school information (Title-1 Status; *r* = 0.15) (all *p* < 0.05). The ARHQ was significantly and negatively correlated with children’s WR skills measured after first (*r* = −0.19) and second grades (*r* = −0.17), as well as with AR abilities captured after first (*r* = −0.14) and second grades (*r* = −0.20) (all *p* < 0.05). The ARHQ was also significantly and negatively correlated with children’s performance on RC tasks administered after second (*r* = −0.18) and third grades (*r* = −0.17), as well as on PS assessment collected after second grade (*r* = −0.26) (all *p* < 0.05).

##### Factor Scores

When unpacking these correlations using factor scores, findings revealed that the Childhood Ability was significantly and negatively associated with children’s WR (*r* = −0.24 and −0.20) and AR skills (*r* = −0.15 and −0.15), as well as performance on RC (*r* = −0.16) and PS tasks (*r* = −0.26) (all *p* < 0.05). The Attitude/Exposure factor was significantly and negatively correlated with children’s performance on RC (*r* = −0.16 and −0.24) and PS performance (*r* = −0.27 and −0.25) (all *p* < 0.05). The Media Use factor was significantly and negatively related to children’s AR skill (*r* = −0.19), as well as performance on RC (*r* = −0.22) and PS tasks (*r* = −0.19) (all *p* < 0.05). The Spelling factor was significantly and negatively linked to children’s WR skill (*r* = −0.16, *p* < 0.05).

#### Familial History of Reading Difficulties

##### Total Scores

Familial history of math difficulties, as indexed by total scores on the AMHQ, was significantly correlated negatively with parents’ educational attainment (*r* = −0.21) and positively with children’s school information (Title-1 Status; *r* = 0.24) (both *p* < 0.05). AMHQ total scores were also significantly and negatively correlated with children’s AR skills measured after first (*r* = −0.17) and second grades (*r* = −0.29) (both *p* < 0.05). These results highlight the criterion validity of the AMHQ in relation to the ARHQ and parents’ educational attainment. Findings also reflect on the construct validity in that the AMHQ is preferentially linked to children’s math- but not reading-related measures (i.e., AR outcomes).

##### Factor Scores

When unpacking these correlations using factor scores, findings revealed that the Childhood Ability factor was significantly ad negatively correlated with children’s WR (*r* = −0.20) and AR skills (*r* = −0.23 and −0.31), as well a performance on PS task (*r* = −0.31) (all *p* < 0.05). The Attitude/Exposure factor was significantly and negatively associated with children’s AR skill (*r* = −0.27, *p* < 0.05).

### Path Findings

**FIGURE 1:**
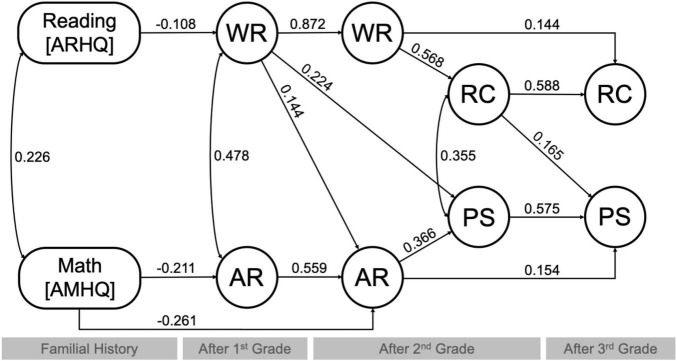
Final path model for the direct and indirect effects of familial history of reading and math difficulties (i.e., indexed by ARHQ and AMHQ, respectively) on measures for children’s core academic skills [word reading (WR) and arithmetic calculation (AR)] after first and second grades, and performance on complex tasks [reading comprehension (RC) and problem solving (PS)] after second and third grades. Parents’ level of educational attainment and children’s demographic information [age, IQ (at baseline), sex, and school information (Title 1 Status)] were included as covariates. (All paths with coefficients shown met *p* < 0.05).

**FIGURE 2:**
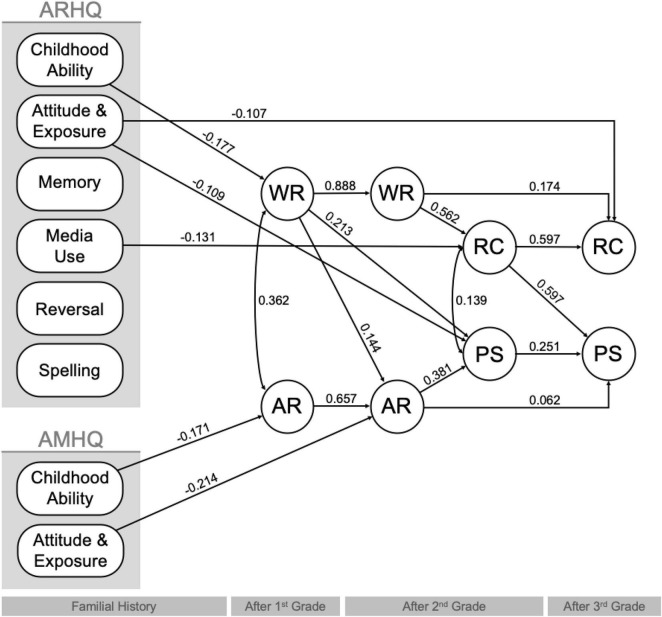
Final path model for the effects of individual factors derived from the ARHQ and AMH on measures of children’s core academic skills [word reading (WR) and arithmetic calculation (AR)] after first and second grades, and performance on complex tasks [reading comprehension (RC) and problem solving (PS)] after second and third grades. Parents’ level of education attainment and children’s demographic information [age, IQ (at baseline), sex, and school information (Title 1 Status)] were included as covariates. (All paths with coefficients shown met *p* < 0.05. Covariances among factors from the ARHQ and AMHQ can be found in Supplementary Table 10. The directionality of the computed factor scores remain consistent with the that of the questions on each scale – that is, higher scores on any factors extracted from the ARHQ or AMHQ indicate, e.g., increased difficulties with learning to read or do simple arithmetic).

Path modeling strategies were employed to evaluate the direct and indirect effects of familial history of reading and math difficulties on children’s core academic skills and performance on complex academic tasks. Variables for familial reading and math history (ARHQ and AMHQ), core academic skills (WR and AR), and performance on complex tasks (RC and PS) reported in correlational findings were subjected to path modeling, with parents’ educational attainment and children’s demographic variables [age, IQ (at baseline), sex, and school information (Title 1 Status)] included as covariates. The initial model (see section “Materials and Methods” and Supplementary Figure 1) was reported with χ^2^ = 58.472 (*p* = 0.000), *CFI* = 0.974, *TLI* = 0.832, *RMSEA* = 0.115 (*p* = 0.002), and *SRMR* = 0.068, indicating a fair fit. To improve model fit, non-significant paths were constrained to zero. The final model was reported with χ^2^ = 82.513 (*p* = 0.002), *CFI* = 0.977, *TLI* = 0.963, *RMSEA* = 0.046 (*p* = 0.369), and *SRMR* = 0.043, indicating a good fit. This step in constraining non-significant paths to zero did not significantly improve the fit (Δχ^2^ = 24.041, *p* = 0.674) when comparing the initial (full) and final models, though the latter was more parsimonious and thus reported here (see Figure 1). Summary findings from the final model can be found in Table 6. Findings from follow-up analyses to distinguish the effects of individual factors derived from the ARHQ and AMHQ on measures of children’s academic performance can be found in Figure 2 and Table 7 and are discussed in Supplementary Results.

**TABLE 6:** Direct and indirect effects of familial history of reading and math difficulties on children’s core academic skills [word reading (WR) and arithmetic calculation (AR)] after first and second grades, and performance on complex academic tasks [reading comprehension (RC) and problem solving (PS)] after second and third grades.

Path	*b*	*se*	*p*

**Direct and indirect effects on core academic skills**			
**Familial reading history (ARHQ)**			
→ WR (1st grade)	**−0.108**	**0.040**	**0.006**
→ WR (1st grade) → WR (2nd grade)	**−0.094**	**0.035**	**0.007**
→ WR (1st grade) → AR (2nd grade)	**−0.016**	**0.006**	**0.000**

**Familial math history (AMHQ)**			
→ AR (1st grade)	**−0.211**	**0.037**	**0.000**
→ AR (2nd grade)	**−0.261**	**0.079**	**0.001**
→ AR (1st grade) → AR (2nd grade)	**−0.118**	**0.019**	**0.000**

**Indirect effects on performance on complex academic tasks**			

**Familial reading history (ARHQ)**			
→ WR (1st grade) → WR (2nd grade) → RC (2nd grade)	**−0.054**	**0.022**	**0.014**
→ WR (1st grade) → WR (2nd grade) → RC (3rd grade)	−0.014	0.010	0.183
→ WR (1st grade) → WR (2nd grade) → RC (2nd grade) → RC (3rd grade)	**−0.032**	**0.013**	**0.013**
→ WR (1st grade) → WR (2nd grade) → RC (2nd grade) → PS (3rd grade)	−0.009	0.003	0.160
→ WR (1st grade) → PS (2nd grade)	−0.024	0.018	0.192
→ WR (1st grade) → PS (2nd grade) → PS (3rd grade)	−0.014	0.003	0.075
→ WR (1st grade) → AR (2nd grade) → PS (2nd grade)	**−0.006**	**0.002**	**0.014**
→ WR (1st grade) → AR (2nd grade) → PS (3rd grade)	−0.002	0.001	0.112
→ WR (1st grade) → AR (2nd grade) → PS (2nd grade) → PS (3rd grade)	**−0.003**	**0.000**	**0.001**

**Familial math history (AMHQ)**			
→ AR (1st grade) → AR (2nd grade) → PS (2nd grade)	**−0.043**	**0.008**	**0.000**
→ AR (1st grade) → AR (2nd grade) → PS (3rd grade)	−0.018	0.004	0.123
→ AR (1st grade) → AR (2nd grade) → PS (2nd grade) → PS (3rd grade)	**−0.025**	**0.003**	**0.003**
→ AR (2nd grade) → PS (2nd grade)	**−0.096**	**0.029**	**0.003**
→ AR (2nd grade) → PS (3rd grade)	−0.040	0.012	0.198
→ AR (2nd grade) → PS (2nd grade) → PS (3rd grade)	**−0.055**	**0.003**	**0.000**

*(Coefficients in bold met p < 0.05).*

**TABLE 7:** Direct and indirect effects of individual factors derived from the ARHQ (centrally, the childhood ability, attitude and exposure, and media use ones) and AMHQ (the childhood ability and attitude and exposure ones) on children’s core academic skills (WR and AR), and performance on complex tasks (RC and PS).

Path	*b*	*se*	*p*

**Direct and indirect effects on core academic skills**			
**Familial reading history (ARHQ)**			
Childhood ability			
→ WR (1st grade)	**−0.177**	**0.052**	**0.001**
→ WR (1st grade) → WR (2nd grade)	**−0.158**	**0.053**	**0.003**
→ WR (1st grade) → AR (2nd grade)	**−0.026**	**0.008**	**0.002**

**Familial math history (AMHQ)**			
Childhood ability			
→ AR (1st grade)	**−0.171**	**0.042**	**0.000**
→ AR (1st grade) → AR (2nd grade)	**−0.112**	**0.025**	**0.000**
Attitude and exposure			
→ AR (2nd grade)	**−0.214**	**0.026**	**0.000**

**Direct and indirect effects on performance on complex academic tasks**			

**Familial reading history (ARHQ)**			
Childhood ability			
→ WR (1st grade) → WR (2nd grade) → RC (2nd grade)	**−0.089**	**0.042**	**0.034**
→ WR (1st grade) → WR (2nd grade) → RC (3rd grade)	−0.027	0.017	0.101
→ WR (1st grade) → WR (2nd grade) → RC (2nd grade) → RC (3rd grade)	**−0.053**	**0.026**	**0.041**
→ WR (1st grade) → WR (2nd grade) → RC (2nd grade) → PS (3rd grade)	−0.007	0.004	0.081
→ WR (1st grade) → PS (2nd grade)	−0.038	0.021	0.069
→ WR (1st grade) → PS (2nd grade) → PS (3rd grade)	−0.009	0.005	0.075
→ WR (1st grade) → AR (2nd grade) → PS (2nd grade)	**−0.010**	**0.003**	**0.001**
→ WR (1st grade) → AR (2nd grade) → PS (3rd grade)	−0.002	0.001	0.064
→ WR (1st grade) → AR (2nd grade) → PS (2nd grade) → PS (3rd grade)	**−0.002**	**0.001**	**0.000**
Media use			
→ RC (2nd grade)	**−0.131**	**0.052**	**0.013**
→ RC (2nd grade) → RC (3rd grade)	**−0.078**	**0.033**	**0.018**
→ RC (2nd grade) → PS (3rd grade)	**−0.010**	**0.005**	**0.045**
Attitude and exposure			
→ RC (3rd grade)	**−0.107**	**0.054**	**0.047**
→ PS (2nd grade)	**−0.109**	**0.052**	**0.036**
→ PS (2nd grade) → PS (3rd grade)	**−0.027**	**0.013**	**0.041**

**Familial math history (AMHQ)**			
Childhood ability			
→ AR (1st grade) → AR (2nd grade) → PS (2nd grade)	**−0.043**	**0.010**	**0.000**
→ AR (1st grade) → AR (2nd grade) → PS (3rd grade)	−0.007	0.003	0.017
→ AR (1st grade) → AR (2nd grade) → PS (2nd grade) → PS (3rd grade)	**−0.011**	**0.003**	**0.000**
Attitude and exposure			
→ AR (2nd grade) → PS (2nd grade)	**−0.081**	**0.008**	**0.000**
→ AR (2nd grade) → PS (3rd grade)	−0.013	0.007	0.068
→ AR (2nd grade) → PS (2nd grade) → PS (3rd grade)	**−0.020**	**0.003**	**0.000**

*(Correlation coefficients in bold met p < 0.05. The directionality of the computed factor scores remain consistent with the that of the questions on each scale – that is, higher scores on any factors extracted from the ARHQ or AMHQ indicate, e.g., increased difficulties with learning to read or do simple arithmetic).*

#### Direct and Indirect Effects of Familial Reading History on Core Academic Skills

##### Total Scores

Familial history of reading difficulties, indexed by total scores on the ARHQ, was shown to have a direct and negative effect on children’s WR skill captured after first grade (*b* = −0.108), which in turn had an indirect effect on WR outcome assessed after second grade (*b* = −0.094) (*p* < 0.05). Interestingly, it appeared that familial history of reading difficulties had an indirect effect on children’s AR outcome measured after second grade via WR skill evaluated after first grade (*b* = −0.016, *p* < 0.05).

##### Factor Scores

Briefly, when unpacking these with factor scores on the ARHQ, findings suggested that the Childhood Ability factor significantly explained the extent to which familial reading history is negatively related to children’s core academic skills.

#### Direct and Indirect Effects of Familial Math History on Core Academic Skills

##### Total Scores

Familial history of math difficulties, indexed by total scores on the AMHQ, was revealed to have direct and negative effects on children’s AR abilities captured after first (*b* = −0.211) as well as second grades (*b* = −0.261), uniquely from autoregressive effects (*p* < 0.05). Familial history of math difficulties was also demonstrated to have an indirect effect on children’s AR outcomes at second via first grades (*b* = −0.118, *p* < 0.05).

##### Factor Scores

When unpacking these effects with factor scores on the AMHQ, the Childhood Ability and Attitude/Exposure factors were both found to explain the extent to which familial math history is negatively related to children’s core math abilities.

#### Indirect Effects on Familial Reading History on Complex Academic Tasks

##### Total Scores

Familial history of reading difficulties, indexed by total scores on the ARHQ, was shown to have an indirect effect on children’s performance on RC task evaluated after second grade via the serial effects of WR skills measured after first and second grades (*b* = −0.054, *p* < 0.05). Such indirect effect of familial history on RC performance captured after second grade in turn had an impact on children’s RC outcome assessed after third grade (*b* = −0.032, *p* < 0.05). Interestingly, familial history of reading difficulties was demonstrated to have an indirect effect on children’s performance on PS task measured after second grade via the serial effects of WR skill captured after first grade on AR ability after second grade (*b* = −0.006, *p* < 0.05). Such indirect effect of familial history on PS performance assessed after second grade thereby had an impact on children’s PS outcome evaluated after third grade (*b* = −0.003, *p* < 0.05).

##### Factor Scores

When unpacking these effects with factor scores on the ARHQ, the Childhood Ability factor was suggested to explain the extent to which familial reading history is indirectly related to children’s performance on complex academic tasks via their core reading skills. The Media Use factor appeared to explain the extent to which familial reading history is directly associated children’s performance on complex academic tasks. The Attitude/Exposure factor was similarly shown to explain the extent to which familial reading history is directly linked to children’s performance on complex academic tasks.

#### Indirect Effects on Familial Math History on Complex Academic Tasks

##### Total Scores

Familial history of reading difficulties, indexed by total scores on the ARHQ, was revealed to have an indirect effect on children’s performance on PS task evaluated after second grade via the serial effects of AR skills measured after first and second grades (*b* = −0.043, *p* < 0.05). This indirect effect of familial history on PS performance captured after second grade in turn had an impact on children’s PS outcome assessed after third grade (*b* = −0.025, *p* < 0.05). Familial history of math difficulties was also demonstrated to have an indirect effect on children’s performance on PS task measured after second grade via AR skill captured also after second grade (*b* = −0.096, *p* < 0.05). The indirect effect of familial history then had an impact on children’s PS outcome evaluated after third grade (*b* = −0.055, *p* < 0.05).

##### Factor Scores

When unpacking these effects with factor scores on the AMHQ, the Childhood Ability factor explained the extent to which familial math history is indirectly associated with children’s performance on complex math tasks via their core math skills. The Attitude/Exposure factor was similar, in that it explained the extent to which familial math history is indirectly associated with children’s performance on complex math tasks via their core math skills.

## Discussion

The current study aimed to characterize the extent to which familial history of academic difficulties was related to children’s reading and math outcomes. Preliminary findings confirmed the psychometric properties of the ARHQ and established the reliability and validity of the AMHQ. Results replicated the negative correlations between scores on the ARHQ and measures of children’s reading outcomes (WR and RC), indicating that heightened familial history of reading difficulties is linked to worse reading performance. Similarly, higher scores on the AMHQ were linked to children’s difficulties in math tasks (AR), thus corroborating the association between familial math history and math outcomes. In terms of the core versus complex pairings of academic outcomes in path analyses, familial history of reading difficulties indirectly explained differences in children’s RC performance via their WR skills, and familial history of math difficulties was indirectly linked to children’s PS outcomes via their AR abilities. Interestingly, scores on the ARHQ were also found to be negatively correlated with measures of children’s math performance (AR and PS), with analyses revealing that these relations were indirectly influenced by differences in their WR skills. The AMHQ had distinctively direct and indirect effects on children’s math performance, but not reading outcomes. Below we further unpack these findings, followed by limitations, potential directions, and implications for this line of research.

### Parents’ Self-Report of Academic Difficulties

#### Adult Reading History Questionnaire

##### Total Scores

Preliminary findings confirmed the psychometric properties for the ARHQ. The ARHQ was reported here with good internal consistency, which is in line with previous studies (Lefly and Pennington, 2000; Pennington and Lefly, 2001). Item-level analyses indicated that questions describing difficulties with learning to read were highly correlated with total scores on the ARHQ. Questions on difficulties with learning to read have been previously reported to be the driving component of the ARHQ as it could flag symptoms of dyslexia, whereas other items in this scale illustrate behavioral features linked to childhood reading differences, including current reading attitude and literacy exposure, that could persist into adulthood (Welcome and Meza, 2019; Feng et al., 2020). Moreover, scores on the ARHQ were shown to be negatively correlated with parents’ reading performance. Interestingly, scores on the ARHQ were also revealed to be negatively associated with parents’ math abilities.

##### Factor Scores

In keeping with past work (Welcome and Meza, 2019), our results supported a six-factor structure for the ARHQ, including Childhood Ability, Attitude/Exposure, Memory, Media Use, Reversal, and Spelling. Childhood Ability factor consisted of items concerning parents’ experiences with learning to read in elementary school, which was shown to be associated with their reading and math skills. Items loading to Attitude/Exposure factor referenced current literacy practices, such as reading for leisure, and attitude toward reading in parents, as well as appeared to be linked with their reading but not math skills. Items making up Memory and Reversal factors contained details about, e.g., “names of places” and “phone numbers,” as well as “letters or numbers,” respectively, so could pertain to both reading and math domains. The Reversal factor, but not Memory, was shown to be correlated with both academic skills among parents from our sample, which is consistent with previous findings on reading (Welcome and Meza, 2019). Items in Media Use factor inquired about parents’ usage of print and media, such as newspapers. Previous studies have suggested that print and media exposure is related to differences in general knowledge and information acquisition (Stanovich and Cunningham, 1993). In our results, the Media factor was not linked to parents’ reading or math skills, which overlaps with prior evidence in reading (Welcome and Meza, 2019). One item loaded into Spelling factor asked parents to contrast their spelling ability to peers of similar age and education, which was demonstrated to be related to parents’ both reading and math skills. This aligns with previous literature as spelling is reliant on phonological processes and linked to comorbid differences in academic abilities (Landerl and Moll, 2010; Slot et al., 2016).

#### Adult Math History Questionnaire

##### Total Scores

The reliability and validity of the AMHQ were also established. Parents’ self-report data on the AMHQ were analyzed with good internal consistency. Similar to findings on the ARHQ, item-level analyses revealed that questions linked to difficulties with completing math work were highly correlated with total scores on the AMHQ. Other items in this scale tapping respondents’ current practices or numeracy exposure were demonstrated with lower correlations with the total scores. Total scores on the AMHQ were shown to be negatively correlated with parents’ math and, to a lesser extent, reading performance. These results confirm the construct validity of the AMHQ by showing its correspondence with parents’ math differences. Additionally, the criterion validity of the AMHQ was supported based on the positive correlation between scores on this scale and the ARHQ. The link between familial history of reading and math difficulties, particularly the Childhood Ability factors from the ARHQ and AMHQ, may also implicate a common feature or underlying cognitive mechanism in learning and academic achievement (Light and DeFries, 1995; Niemi et al., 2011; Costa et al., 2013; Fletcher et al., 2019).

##### Factor Scores

Further exploratory analyses suggested a two-factor structure for the AMHQ, which reflected Childhood Ability and Attitude/Exposure. Consistent with first two factors in the ARHQ that query about the respondents’ childhood and current reading experiences, Childhood Ability factor in the AMHQ included items about parents’ self-reported difficulties with learning math contents in elementary education, and Attitude/Exposure factor surveyed current numeracy practices and attitude toward math. The Childhood Ability factor from each scale was shown to be correlated with parents’ both reading and math skills. Whereas Attitude/Exposure factor from the ARHQ appeared to be uniquely associated with reading abilities in parents, this factor from the AMHQ was linked to their math proficiency. On the other hand, not only were a couple of remaining factors from the ARHQ were limited to one item, particularly Reversal and Spelling, these and others, including Memory and Media Use, did not appear to have an analogous AMHQ factor. Scores on the Reversal and Spelling dimensions from ARHQ, as aforementioned, were correlated with differences in parents’ both reading and math skills. Despite differences in the numbers of factors found for each scale (six for ARHQ versus two for AMHQ), the patterns of associations between parents’ academic functioning and these factors implicate overlapping or cross-domain effects (Childhood Ability, Reversal, and Spelling), as well as unique roles in either reading or math (Attitude/Exposure).

### Familial History of Reading Difficulties

#### Total Scores

Correlational analyses were consistent with previous reports that have shown that familial history of reading difficulties negatively predicts differences in children’s reading outcomes. Higher scores on the ARHQ were indeed associated with children’s decreased ability to read individual words (WR skills), as well as worse performance on tasks that asked them to read, connect, and comprehend text using a cloze format (RC). Using samples of preschool children or those starting formal education, previous studies have reported the negative relations between familial history of reading difficulties and differences in children’s emergent literacy skills, basic reading abilities (i.e., WR), and text reading fluency outcomes (e.g., Carroll and Snowling, 2004; Giménez et al., 2017). Some prior studies have observed a link between familial reading history and children’s RC (Pennington and Lefly, 2001). Altogether, parents’ self-report of increased reading difficulties could signal differences and degree of difficulty and severity in children’s reading achievement. Some reports have further unpacked how familial history of reading difficulties is linked to reading over development, e.g., indirectly to RC via WR (Hulme et al., 2015).

The negative association between familial history of reading difficulties and children’s performance on complex reading tasks (RC) was shown to be indirectly predicted by differences in core reading skills. These findings are not altogether surprising as previous reports have shown that familial history of reading difficulties has a negative impact on children’s emergent literacy skills, and that this association in turn has an effect on their WR abilities that are measured when they start receiving explicit reading instruction (e.g., Esmaeeli et al., 2019). The current study built on these prior results by showing that familial history of reading difficulties has an indirect effect on children’s RC performance by tapping their core reading skills (WR), which is consistent with other results also using a longitudinal design and path analyses (Hulme et al., 2015).

#### Factor Scores

When unpacking which factors within the ARHQ were driving the path findings in our study, two following patterns emerged: Childhood Ability was correlated with core reading skills, whereas Attitude/Exposure and Media Use were associated with differences in complex reading performance. The Childhood Ability dimension appeared to explain the direct effect of familial reading history on children’s WR skills, which in turn had an impact on their RC performance. These findings hold true even after adjusting for children’s IQ (a combination of both verbal and non-verbal subscales). This in part supports the hypothesis for the familial influence on basic reading skills, which prior literature suggests would be at least driven by phonological abilities, in children’s reading development (Pennington, 2006; van Bergen et al., 2014). Notably, the Attitude/Exposure and Media Use factors from the ARHQ were shown to have unique effects on children’s RC performance, which was independent from and not indirectly through their WR skills. These findings are congruent with previous findings (Welcome and Meza, 2019) and suggest a link between parents’ own attitude toward reading and literacy practices and children’s performance on complex reading tasks.

### Familial History of Math Difficulties

#### Total Scores

Findings showed that the familial history of math difficulties was negatively correlated with children’s math outcomes. Higher scores on the AMHQ were found to be associated with children’s lower performance in solving simple arithmetic tasks (addition/subtraction; AR). This is consistent with previous findings that used either the dichotomous self-report questionnaire as well as those that used performance-based measures collected from parents to operationalize their difficulties with arithmetic and computing skills (Shalev et al., 2001; Khanolainen et al., 2020; see also Wijsman et al., 2004). These findings also substantiate the construct validity of the AMHQ by tracking its link with children’s math outcomes. Notably, using path analyses, the direct predictive effects of the AMHQ were shown in children’s levels of AR performance assessed after both first and second grades, where the stability of individual differences in such math skills between these two occasions (or autoregressive effect) was represented. These findings suggest that familial history of math difficulties not only impacts initial AR abilities, but also predicts AR growth. Moreover, scores on the AMHQ were not directly related to children’s performance on applied math problems. Instead, familial history of math difficulties was shown to have an indirect effect on children’s performance on complex math tasks (PS) entirely via their core math skills (AR).

##### Factor Scores

Supplementary analyses revealed that the Childhood Ability and Attitude/Exposure features of familial math history, as derived from the AMHQ, explained the extent to which parents’ self-report of math difficulties indirectly related to children’s PS performance through their AR skills. Previous literature has indicated the links between parents’ dichotomous self-report of math difficulties and children’s arithmetic and computing skills (Khanolainen et al., 2020), as well as between these core math abilities and performance on complex problem-solving tasks in children (Fuchs et al., 2006). These prior findings are consistent with our findings for an indirect prediction of familial math history in children’s PS outcome via their AR skills (Bauer et al., 2006). Furthermore, some reports examining the intergenerational transmission of math difficulties have observed substantial correspondence in pre-numeracy abilities (e.g., approximate number system) between parents and children (Braham and Libertus, 2017; Bernabini et al., 2021). Familial history of math difficulties likely plays a role in children’s pre-numeracy abilities before formal education, as well as their arithmetic skills that are introduced through explicit classroom instruction. On the other hand, the non-significant direct effect of familial history of math difficulties on children’s performance on complex math tasks (PS) may be less surprising than expected because of additional cognitive processes involved and/or the use of specific language to teach math concepts and assess the respective understanding (e.g., Fuchs et al., 2006, 2021). Complex math tasks have been shown to place demands on not just AR skills but also non-verbal reasoning, concept formation, executive function, oral language, and WR abilities (Fuchs et al., 2006; Spencer et al., 2020). Some reports have conjectured that difficulties in children’s performance on complex math tasks at the arithmetic level might be offset or compensated by some of these cognitive processes, such as oral language (Fuchs et al., 2018).

### Comorbid Reading and Math Difficulties

#### Total Scores

Consistent with previous findings, familial history of reading difficulties was found to be negatively associated with children’s math outcomes, while familial history of math difficulties was not linked to their reading performance. For example, Pennington and Lefly (2001) found evidence for the relations between scores on the ARHQ and children’s AR skills as well as performance on PS tasks (Pennington and Lefly, 2001). Children with familial history of reading difficulties are thought to in part face challenges in meeting the verbal demands in math tasks (Khanolainen et al., 2020), given that previous theoretical accounts have posited a unique role for linguistic processes in math performance (e.g., Triple Code Model, see Dehaene and Cohen, 1995; Abstract-Code Model, see McCloskey, 1992). As aforementioned, phonological awareness is a known predictor in reading development (Storch and Whitehurst, 2002; Cirino et al., 2018), and has been shown to be linked to scores on the ARHQ (Pennington and Lefly, 2001). Studies suggested that phonological awareness is also a predictor of children’s math performance (e.g., Slot et al., 2016; Child et al., 2019; Amland et al., 2021), perhaps more so in AR skills than PS outcomes (see Fuchs et al., 2006). Together these findings could be taken to mean that familial history of reading difficulties plays a role in children’s reading and math outcomes via phonological or verbal processes. It is worthy to note, however, that math performance also draws on unique skills that are not necessarily tied to processes in reading; one skill that distinguishes math from reading is the approximate number system (Slot et al., 2016; Cirino et al., 2018), which some have previously reported as being linked to familial history of math difficulties (Braham and Libertus, 2017; Bernabini et al., 2021). The relation between familial history of math difficulties and children’s math outcomes could thus be distinctly tapping skills, such as the approximate number system, that are not predictors of reading outcomes. This premise could explain the current results of the non-significant link between the AMHQ and measures of children’s reading performance.

The relation between familial history of reading difficulties and children’s math performance was shown to be facilitated by differences in children’s core reading skills, as scores on the ARHQ were indirectly linked to children’s AR abilities via their WR skills. Although not within the framework of familial history, recent longitudinal studies on comorbid academic differences have demonstrated that early reading skills are predictive of later math outcomes, but not vice versa, as their associations unfold over the first years of formal education (Erbeli et al., 2019). The current study builds on these findings by suggesting that the impact of familial reading history on children’s reading performance could have downstream effect on their math abilities. Some reports have interpreted that the relation between familial history of academic difficulties and comorbid reading and math differences in children could signal a procedural learning problem or an inadequate response to instruction (Light and DeFries, 1995; Niemi et al., 2011). Furthermore, the extent to which familial history of reading difficulties had a negative impact on children’s WR and in turn AR skills appeared to subsequently relate to their performance on PS tasks. These findings are in line with those that have shown that compared to peers with inadequate math skills, children with comorbid reading problems are more likely struggle with PS tasks of varying complexity (Fuchs and Fuchs, 2002). Findings from the current study suggest that familial history of reading difficulties could exacerbate the comorbid differences in children’s reading and math skills, which could then result in poor performance on complex math tasks.

##### Factor Scores

Follow-up findings indicate that specific factors in familial reading history, as derived from the ARHQ, were differentially related to levels of children’s academic outcomes: Childhood Ability predicted core reading skills and in turn permeated math outcomes, whereas Attitude/Exposure and Media Use were linked to performance on complex reading and math tasks. Association between parents’ difficulties with learning to read in elementary school and children’s abilities in core academic skills (WR and AR) could indicate some common phonological and verbal processes, as well as differences with procedural learning performance (e.g., Light and DeFries, 1995; Niemi et al., 2011; Child et al., 2019; Amland et al., 2021). What is particularly interesting were the direct effects of the Attitude/Exposure factor from the ARHQ on children’s performance on both complex reading and math tasks (RC and PS). And, the Media Use factor was demonstrated to directly have an impact children’s RC performance and in turn indirectly on PS outcomes. Together these findings could be taken to mean that parents’ literacy practices (reading newspapers and/or books for leisure) are related to some core cognitive components, other than WR skills, that are key to performance on both complex reading and math tasks. In contrast, familial history of math difficulties was not shown to have any substantial effects on children’s reading outcomes directly, or indirectly via their math skills. These findings implicate that there is a unique role for familial math history in children’s math outcomes, versus the more ubiquitous impact of familial reading history on both reading and math performance in children.

### Limitations and Alternative Considerations

While the current findings offer novel insights into the role of familial history of reading and math difficulties, it is not without limitations. Parents’ self-report was used as a way to survey familial history of academic difficulties. Within the past decades, studies have found the ARHQ useful in characterizing whether a child is at risk for difficulties with reading development (or dyslexia; e.g., Black et al., 2012; Rosenberg et al., 2012); the current report showed that both the ARHQ and AMHQ were related to parents’ reading and math performance, respectively. As with any self-report measures, concerns remain in regard to the credibility of parents’ endorsement on a questionnaire about their retrospective learning experiences in school, interpretation of “difficulty” when learning to read versus math concepts, and perception of own versus peers’ performance (e.g., “… skill compared to others”) across academic domains.

The psychometric findings on the ARHQ and AMHQ prompted a consideration for their dimensionality. While total scores on the ARHQ were previously used to capture the continuous nature of familial reading history, the wide range of correlation coefficients among items within this survey denotes presence of more than one dimension (as reported here, *r*’s = between −0.115 and 0.809). Findings from previous and our work indeed discern specific dimensions in the ARHQ, notably Childhood Ability and Attitude/Exposure (Welcome and Meza, 2019). In terms of the AMHQ, its items included different phrasing – i.e., in the forms of a question or statement – and also displayed a wide range of inter-item correlation coefficients (*r*’s = between 0.127 and 0.857). It should be noted, however, that items within the AMHQ loaded highly into corresponding constructs, Childhood Ability versus Attitude/Exposure, derived from this scale. These results not only suggest that the factor findings were not driven by the different phrasing, but also familial math history could be distinguished into specific dimensions. Future studies may take into account the continuous *and* dimensional nature of familial academic history by utilizing total and factor scores on the ARHQ and AMHQ.

Follow-up work should consider further examining the psychometric and predictive properties of the ARHQ and AMHQ, where the latter scale queries the respondents’ experiences with math content generally, alongside with other academic history questionnaires, such as the *Adult Arithmetic Questionnaire* (AAHQ; Sury and Gaab, 2020). While the AAHQ has yet to be validated, it should be noted that this survey delves into the more granular components of math than the AMHQ (e.g., math facts, counting and estimation, memory for numbers, problem solving, simple and complex arithmetic); inclusion of such items may prove to have additional predictive power in children’s outcomes. Notably, items in the ARHQ asked about the respondents’ overall experience with reading rather than its specific components (e.g., rapid naming, phonological awareness, single word versus passage reading efficiency, and reading comprehension), which prompted our decision for the AMHQ to ask broad questions about math in a parallel format and not assess subcomponents. Further investigations of parents’ reading experiences may consider emulating the nuanced strategies adopted in the AAHQ to tackle these various levels of reading performance, as well as to look at the familial learning history in terms of academic subcomponents.

Given the focus on investigating familial academic history, outcomes measures of interest were children’s core versus complex reading and math skills. While the current findings offer some insights for the role of familial academic history in comorbid academic difficulties, future studies should consider nuanced genetic or twin design, along with the ARHQ and AMHQ, and in large-scale samples to create adequate grouping of children with single or combined deficits in reading and/or math (as executive in, e.g., Erbeli et al., 2019). It should be noted that children’s performance on complex reading and math tasks is known to draw on cognitive processes other than core academic skills, including oral language and executive function. Proficiency in oral language is key to both RC and PS outcomes because of the verbal and linguistic demands in performance on these complex tasks (Child et al., 2019). Meta-analytic findings have revealed that familial history of reading difficulties is negatively associated with children’s oral language proficiency, which could undermine their reading development (Snowling and Melby-Lervåg, 2016). Other reports have posited that intact oral language processes could play a compensatory role in RC among children with heightened familial history of reading difficulties (Torppa et al., 2007). Executive function, e.g., working memory, is another cognitive predictor of children’s performance on complex reading and math tasks (Fuchs et al., 2006; Cutting et al., 2009; Cirino et al., 2018). Furthermore, intergenerational transmission of math anxiety could be linked to differences in children’s executive function and performance on math tasks (Chang and Beilock, 2016). Therefore, to fully unpack the nature of the association between familial history and academic outcomes, future studies could consider examining cognitive predictors of children’s reading and math outcomes, as well as math anxiety, in relation to familial history of academic difficulties (and factors derived from the ARHQ and AMHQ).

## Summary and Implications

The current study provides results that are promising both theoretically and practically, with implications for children at heightened familial history for academic difficulties. First, the novel simultaneous integration of the ARHQ and AMHQ suggests that assessing familial risk for academic difficulties may be important for understanding comorbid etiological and developmental associations among children’s academic outcomes. In particular, findings on the roles of familial reading history and children’s reading abilities in their math outcomes supply evidence for the phonological pathway in these academic domains (Geary, 1993; Child et al., 2019; Amland et al., 2021). This hypothesis on the phonological pathway may elicit the consideration of incorporating literacy contents in classroom instruction and interventions focusing on numeracy materials. Some reports have suggested that children with single or comorbid difficulties in reading and/or math could benefit from some forms of combined reading and math remediations (e.g., Fuchs et al., 2012; Glenberg et al., 2012; Powell et al., 2020).

Second, utility of parents’ self-report information on the ARHQ and AMHQ as additional diagnostic metrics could facilitate early identification of children who are at heightened risk for reading and/or math difficulties and identify prevention strategies, which could be more effective than to implement later remediation (for intervention findings with known status of familial reading risk, see Muter and Snowling, 2009; Zijlstra et al., 2021). This is also because learning differences are commonly diagnosed not until after children have well entered formal education and exhibited substantial performance difficulties in the classroom – with concerns among many individuals often overlooked or recognized with delay (Fletcher et al., 2019). Some studies have utilized a dichotomous, or yes-versus-no, measure on parents’ general self-report of reading or math difficulties to operationalize familial academic history (Landerl and Moll, 2010; Erbeli et al., 2019; Khanolainen et al., 2020). Using such approach, some have found that this indicator of familial reading history does not contribute substantially beyond performance-based assessment to screening children for reading difficulties (Ferrer et al., 2021). Others have used the ARHQ to capture the continuous nature in familial history of reading difficulties based on related clinical and additive features observed across the lifespan, such as learning to read in elementary school, current reading behaviors and print exposure, and attitude toward literacy (e.g., Lefly and Pennington, 2000). Previous and our work suggests that dimensions within the ARHQ on Childhood Ability, or learning in early education, and Attitude/Exposure, or current practices and interest, map onto the respondents’ academic functioning (for samples of college-aged individuals, see Parilla et al., 2007; Welcome and Meza, 2019; see also Kirby et al., 2008) as well as their children’s outcomes (shown among elementary students here in parallel with findings from the AMHQ; for a sample of adolescents, see also Conlon et al., 2006). Future prediction and preventive studies may want to consider the early learning and current behavioral features in familial reading and math history. For example, parents could be queried about own experiences to gage at their children’s learning potentials; these children as they advance in post-secondary education may be asked about their attitude, interest, and perception toward reading; or individuals in adulthood could be surveyed to identify ways to target specific academic abilities (core skills versus performance on complex tasks; e.g., leisure reading and print exposure).

Third, results regarding the intergenerational effects of familial academic history on children’s academic outcomes point to the contribution of parents’ educational circumstances, literacy and numeracy practices, and role in the home cognitive environment (van Bergen et al., 2014). For example, parents who experienced more difficulties with learning to read in elementary school tend to read less in adulthood (Muter and Snowling, 2009; Snowling and Melby-Lervåg, 2016), and may in turn deliver a less sufficient home literacy environment or promote less reading opportunities for their children (Hamilton et al., 2016). One may also speculate that parents who struggled more with learning math contents in elementary could face more challenges with math materials in adulthood, and perhaps would offer less numeracy practices for their children (Bernabini et al., 2020). Findings for the respective effects of the Childhood Ability factors from the ARHQ and AMHQ on children’s core reading versus math outcomes could implicate some underlying degree of intergenerational mediation or heritability in difficulties when learning to read words or do simple arithmetic (Pennington, 2006). On the other hand, what was shown to be independent from the effects between the Childhood Ability factors from these scales and children’s core academic skills is the unique role of the Attitude/Exposure factor, as well as the Media Use one to some extent, from the ARHQ in their performance on both complex reading and math tasks. These results highlight the distinguishable impacts between parents’ current literacy practices versus their retrospective difficulties with procedural learning (e.g., to read or do simple arithmetic) children’s academic outcomes. Insights from the hypothesis on the intergenerational pathway could encourage future research and intervention efforts to place additional focus on adults’ academic backgrounds and practices, which may confer downstream effects on their offsprings’ educational needs and classroom performance.

## Data Availability Statement

Data in the current study are not publicly available due to not all participants providing consent for their data to be shared outside of Vanderbilt University and Vanderbilt University Medical School. A subset of this data, from those participants who did provide consent for their data to be shared outside of the aforementioned institutions, are available from the corresponding author on reasonable request.

## Ethics Statement

The studies involving human participants were reviewed and approved by Vanderbilt University’s Institutional Review Board. Written informed consent to participate in this study was provided by the participants’ legal guardian/next of kin.

## Author Contributions

TN and AM-L conducted analyses and prepared manuscript draft. LC contributed to funding acquisition, data curation, and supervision. All authors contributed to experimental design, discussing results, revision, and approval of the manuscript.

## Conflict of Interest

The authors declare that the research was conducted in the absence of any commercial or financial relationships that could be construed as a potential conflict of interest.

## Publisher’s Note

All claims expressed in this article are solely those of the authors and do not necessarily represent those of their affiliated organizations, or those of the publisher, the editors and the reviewers. Any product that may be evaluated in this article, or claim that may be made by its manufacturer, is not guaranteed or endorsed by the publisher.

## Footnotes

1 Validated with current parental academic functioning (see Supplementary Table 8).
