# Supplementary material for: Tracking Familial History of Reading and Math Difficulties in Children’s Academic Outcomes
Source: Front Psychol. 2022 Jan 18;12:710380. doi: 10.3389/fpsyg.2021.710380 (PMC8803642; doi:10.3389/fpsyg.2021.710380)
Supplement: Supplementary file 1 [file Data_Sheet_1.pdf]

1. Supplementary Tables

1.1. **Supplementary Table 1.** Descriptive information on individual items from the *Adult Reading History Questionnaire* (Lefly & Pennington, 2000).

Item	Description	*m*	*sd*	Item-Total *r*	Item-Rest *r*	α
**Adult Reading History Questionnaire**						
1	Which of the following most nearly describes your attitude toward school when you were a child?	1.023	0.929	0.455	0.431	0.870
2	How much difficulty did you have learning to read in elementary school?	0.693	1.129	0.727	0.651	0.862
3	How much extra help did you need when learning to read in elementary school?	0.695	1.134	0.668	0.591	0.864
4	Did you ever reverse the order of letters or numbers when you were a child?	0.436	0.897	0.451	0.373	0.871
5	Did you have difficulty learning letter and/or color names when you were a child?	0.173	0.521	0.550	0.494	0.870
6	How would you compare your reading skill to that of others in your elementary classes?	1.087	1.141	0.701	0.641	0.863
7	All students struggle from time to time in school. In comparison to others in your classes, how much did you struggle to complete your work?	1.148	1.029	0.689	0.637	0.863
8	Did you experience difficulty in high school or college English classes?	0.791	0.972	0.660	0.602	0.865
9	What is your current attitude toward reading?	0.349	0.637	0.507	0.472	0.870
10	How much reading do you do for pleasure?	1.319	1.069	0.444	0.388	0.871
11	How would you compare your current reading speed to that of others of the same age and education?	1.411	0.948	0.579	0.548	0.866
12	How much reading do you do in conjunction with your work? (If retired or not working, how much did you read when you were working?)	1.087	1.029	0.401	0.381	0.871
13	How much difficulty did you have learning to spell in elementary school?	0.982	1.167	0.655	0.586	0.864
14	How would you compare your current spelling to that of others of the same age and education?	1.255	1.013	0.587	0.544	0.866
15	Did your parents ever consider having you repeat any grades in school due to academic failure (not illness)?	0.224	0.642	0.467	0.418	0.871
16	Do you ever have difficulty remembering people's names or names of places?	1.480	1.196	0.440	0.407	0.871
17	Do you ever have difficulty remembering addresses, phone numbers, or dates?	1.219	1.151	0.431	0.402	0.871
18	Do you have difficulty remembering complex verbal instructions?	1.005	1.030	0.463	0.430	0.870
19	Do you currently reverse the other of letters or numbers when you read or write?	0.352	0.823	0.461	0.408	0.870
20	How many books do you read for pleasure each year?	1.418	1.197	0.509	0.450	0.869
21	How many magazines do you read for pleasure each month?	2.594	1.104	0.217	0.213	0.876
22	Do you read daily (Monday-Friday) newspapers?	2.253	1.377	0.362	0.335	0.874
23	Do you read a newspaper on Sunday?	2.605	1.228	0.246	0.219	0.877

1.2. **Supplementary Table 2.** Descriptive information on individual items from the *Adult Math History Questionnaire*, which was adapted from the *Adult Reading History Questionnaire* (Lefly & Pennington, 2000).

**Item**	**Description**	***m***	***sd***	**Item-Total *r***	**Item-Rest *r***	**α**
**Adult Math History Questionnaire**						
1	When in elementary school, I struggled with learning new concepts in math.	0.957	1.108	0.763	0.722	0.920
2	When in elementary school, I needed extra help in math from a teacher or tutor.	0.695	1.076	0.677	0.629	0.922
3	How would you compare your math skills to those of others in your elementary classes?	1.246	1.047	0.743	0.712	0.920
4	Compared to others in your elementary classes, how much did you struggle to complete your math work?	0.982	1.121	0.809	0.769	0.918
5	During high school or college, I struggled in math courses.	1.551	1.268	0.778	0.754	0.919
6	I took math classes in high school or college that were not required because I enjoyed them.	2.810	1.410	0.532	0.514	0.927
7	What is your current attitude towards math?	1.066	1.035	0.755	0.724	0.920
8	Math makes me feel uncomfortable and nervous.	1.056	1.034	0.852	0.824	0.917
9	As an adult, I struggle to complete math-related tasks, such as calculating tips.	0.700	0.900	0.705	0.676	0.921
10	Math is important in everyday life.	0.449	0.755	0.470	0.433	0.926
11	My current work requires I use math.	1.285	1.190	0.422	0.375	0.929
12	I enjoy completing math and logic puzzles for fun.	1.867	1.270	0.636	0.608	0.923
13	I use math in my everyday life.	0.715	0.919	0.567	0.519	0.925
14	How would you compare your current math skills compared to those of others of the same age and education?	1.579	0.919	0.750	0.728	0.920
15	I would like to further develop my math skills.	1.711	1.081	0.393	0.371	0.928
16	I feel confident in helping my child with their math homework.	0.893	0.958	0.688	0.662	0.922
17	New math content has usually been easy and enjoyable for me to understand.	1.673	1.079	0.801	0.781	0.918

1.3. **Supplementary Table 3.** Pairwise correlations among items within the *Adult Reading History Questionnaire*. (*Note*. Correlation coefficients in **bold** met $p < 0.05$.)

	Adult Reading History Questionnaire											
**Item**	**1**	**2**	**3**	**4**	**5**	**6**	**7**	**8**	**9**	**10**	**11**	**12**
**Adult Reading History Questionnaire**												
1	-											
2	**0.452**	-										
3	**0.398**	**0.787**	-									
4	0.092	**0.358**	**0.335**	-								
5	**0.168**	**0.533**	**0.472**	**0.526**	-							
6	**0.373**	**0.709**	**0.640**	**0.358**	**0.476**	-						
7	**0.398**	**0.639**	**0.584**	**0.314**	**0.474**	**0.638**	-					
8	**0.309**	**0.558**	**0.517**	**0.320**	**0.462**	**0.595**	**0.476**	-				
9	**0.222**	**0.318**	**0.277**	**0.161**	**0.271**	**0.284**	**0.283**	**0.388**	-			
10	**0.180**	**0.171**	**0.115**	0.062	**0.145**	**0.226**	0.126	**0.241**	**0.564**	-		
11	**0.239**	**0.388**	**0.386**	**0.258**	**0.286**	**0.453**	**0.381**	**0.354**	**0.373**	**0.319**	-	
12	**0.282**	**0.136**	**0.175**	0.053	0.100	**0.178**	**0.148**	**0.235**	**0.309**	**0.217**	**0.322**	-
13	**0.310**	**0.645**	**0.546**	**0.494**	**0.380**	**0.568**	**0.571**	**0.464**	**0.208**	0.034	**0.355**	**0.170**
14	**0.307**	**0.480**	**0.448**	**0.277**	**0.254**	**0.439**	**0.455**	**0.407**	**0.195**	0.133	**0.407**	**0.253**
15	**0.223**	**0.446**	**0.543**	0.068	**0.355**	**0.411**	**0.448**	**0.464**	**0.186**	0.058	**0.251**	0.123
16	**0.205**	**0.146**	0.108	**0.190**	0.125	0.122	**0.256**	0.119	0.092	**0.164**	**0.168**	**0.230**
17	**0.178**	**0.186**	0.083	**0.154**	**0.167**	**0.182**	**0.268**	0.130	0.108	**0.170**	**0.209**	0.134
18	**0.185**	**0.172**	**0.186**	0.111	**0.180**	**0.155**	**0.322**	**0.279**	**0.150**	0.105	**0.187**	**0.314**
19	0.119	**0.332**	**0.286**	**0.573**	**0.223**	**0.296**	**0.300**	**0.353**	0.104	0.056	**0.293**	0.070
20	**0.265**	**0.255**	**0.214**	0.051	**0.191**	**0.285**	**0.169**	**0.261**	**0.537**	**0.809**	**0.345**	**0.278**
21	-0.026	0.028	-0.002	-0.028	-0.004	0.015	0.045	0.081	0.131	**0.184**	0.025	**0.170**
22	0.116	0.064	0.048	-0.050	0.074	0.119	**0.162**	0.115	**0.170**	**0.222**	**0.203**	**0.219**
23	-0.014	-0.093	-0.056	-0.057	0.015	0.006	-0.004	0.041	0.126	**0.175**	**0.156**	**0.255**
ARHQ Total	**0.489**	**0.706**	**0.654**	**0.433**	**0.527**	**0.696**	**0.684**	**0.652**	**0.512**	**0.455**	**0.593**	**0.439**

	Adult Reading History Questionnaire										
**Item**	**13**	**14**	**15**	**16**	**17**	**18**	**19**	**20**	**21**	**22**	**23**
**Adult Reading History Questionnaire**											
13	-										
14	**0.709**	-									
15	**0.300**	**0.241**	-								
16	**0.242**	**0.148**	0.029	-							
17	**0.236**	**0.197**	0.081	**0.652**	-						
18	**0.258**	**0.188**	**0.196**	**0.583**	**0.472**	-					
19	**0.399**	**0.266**	**0.281**	**0.209**	**0.217**	**0.294**	-				
20	0.110	**0.187**	0.126	**0.182**	**0.187**	**0.141**	0.062	-			
21	-0.002	0.123	0.043	**0.152**	**0.167**	0.132	0.033	0.134	-		
22	0.045	**0.158**	0.077	**0.192**	**0.225**	**0.152**	0.027	**0.258**	**0.423**	-	
23	-0.115	-0.008	0.034	**0.231**	0.122	**0.147**	0.066	**0.188**	**0.423**	**0.668**	-
ARHQ Total	**0.644**	**0.591**	**0.477**	**0.482**	**0.472**	**0.485**	**0.458**	**0.526**	**0.286**	**0.419**	**0.314**

1.4. Supplementary Table 4. Pairwise correlations between items from the *Adult Reading History Questionnaire* (columns) and those from the *Adult Math History Questionnaire* (rows). (*Note*. Correlation coefficients in **bold** met $p < 0.05$.)

	**Adult Reading History Questionnaire**											
**Item**	**1**	**2**	**3**	**4**	**5**	**6**	**7**	**8**	**9**	**10**	**11**	**12**
**Adult Math History Questionnaire**												
1	**0.287**	**0.380**	**0.314**	**0.138**	**0.210**	**0.267**	**0.534**	**0.199**	0.129	0.015	0.114	0.012
2	**0.303**	**0.381**	**0.328**	**0.220**	**0.281**	**0.273**	**0.512**	**0.251**	0.118	-0.003	**0.137**	-0.020
3	**0.337**	**0.375**	**0.387**	**0.167**	**0.186**	**0.426**	**0.604**	**0.196**	0.123	0.064	**0.248**	0.006
4	**0.358**	**0.400**	**0.390**	**0.176**	**0.222**	**0.348**	**0.604**	**0.182**	**0.150**	0.018	**0.185**	0.021
5	**0.215**	**0.279**	**0.275**	0.098	**0.172**	**0.210**	**0.457**	0.096	0.065	-0.009	0.087	-0.002
6	0.116	0.078	0.044	0.075	0.039	0.073	**0.202**	-0.067	0.029	0.038	-0.018	0.061
7	**0.141**	0.094	0.019	0.082	0.024	-0.010	**0.173**	-0.065	-0.019	0.007	-0.135	0.068
8	**0.168**	**0.227**	**0.166**	**0.150**	**0.145**	**0.144**	**0.371**	0.027	0.064	-0.027	0.098	0.066
9	**0.165**	**0.269**	**0.274**	**0.197**	**0.206**	**0.297**	**0.422**	**0.148**	0.120	0.001	**0.173**	0.025
10	**0.166**	0.062	0.098	0.109	0.057	0.110	**0.193**	0.032	0.023	-0.030	-0.126	0.103
11	0.117	**0.138**	0.113	0.064	0.100	**0.169**	**0.186**	-0.030	0.067	-0.072	0.093	**0.219**
12	**0.181**	0.034	0.054	0.074	0.084	0.079	**0.207**	0.032	0.061	0.002	-0.047	0.099
13	**0.234**	**0.148**	**0.156**	0.091	**0.149**	**0.212**	**0.287**	0.082	0.114	-0.021	0.047	**0.331**
14	**0.188**	**0.254**	**0.237**	0.084	0.048	**0.229**	**0.367**	0.018	0.018	-0.051	**0.147**	0.046
15	**0.157**	-0.008	0.008	0.066	-0.020	0.031	0.107	-0.028	0.062	0.058	-0.005	**0.220**
16	**0.268**	**0.246**	**0.225**	**0.166**	**0.139**	**0.226**	**0.406**	**0.177**	0.092	0.036	0.117	0.079
17	**0.250**	**0.220**	**0.198**	0.044	0.110	**0.204**	**0.400**	0.115	0.096	-0.003	**0.145**	0.068
AMHQ Total	**0.317**	**0.309**	**0.279**	**0.176**	**0.195**	**0.278**	**0.520**	0.130	0.125	0.013	0.106	0.111

	Adult Reading History Questionnaire										
**Item**	**13**	**14**	**15**	**16**	**17**	**18**	**19**	**20**	**21**	**22**	**23**
**Adult Math History Questionnaire**											
1	**0.328**	**0.225**	**0.326**	**0.154**	**0.230**	**0.213**	**0.139**	0.096	-0.035	0.097	-0.086
2	**0.367**	**0.249**	**0.358**	0.072	**0.182**	**0.162**	**0.165**	0.111	-0.041	0.075	-0.023
3	**0.364**	**0.287**	**0.316**	0.081	**0.143**	0.111	**0.158**	**0.147**	-0.042	0.068	-0.086
4	**0.379**	**0.254**	**0.302**	**0.179**	**0.232**	**0.246**	**0.165**	0.123	-0.032	0.117	-0.037
5	**0.221**	0.092	**0.243**	**0.146**	**0.219**	**0.162**	0.080	0.061	-0.013	0.051	-0.090
6	0.011	0.005	0.080	0.017	**0.150**	0.040	0.036	0.048	0.065	0.123	-0.010
7	0.084	0.024	0.086	0.109	0.124	0.068	0.004	-0.014	-0.043	-0.025	-0.052
8	**0.177**	0.092	**0.188**	0.113	**0.166**	**0.139**	0.127	0.025	0.032	0.019	-0.026
9	**0.204**	**0.155**	**0.277**	**0.161**	**0.269**	**0.264**	**0.198**	0.050	0.002	0.058	-0.020
10	0.106	0.111	0.032	-0.013	0.010	0.000	0.003	0.050	-0.021	-0.015	-0.061
11	0.072	**0.145**	0.084	-0.074	-0.029	0.085	0.034	-0.037	-0.039	0.031	-0.023
12	0.029	0.045	**0.201**	0.078	0.048	0.092	0.057	-0.031	0.128	0.021	0.057
13	0.131	**0.145**	0.123	0.033	0.036	0.126	-0.030	0.004	0.046	0.070	0.020
14	**0.193**	**0.211**	**0.208**	0.082	0.111	0.135	0.095	-0.020	0.043	0.076	-0.024
15	-0.052	0.034	0.001	-0.050	-0.027	-0.063	-0.018	0.092	**0.157**	**0.141**	0.043
16	0.135	**0.167**	**0.290**	0.123	0.060	**0.184**	**0.158**	0.038	0.033	0.036	0.019
17	0.137	**0.177**	**0.217**	0.091	**0.162**	**0.174**	0.055	0.062	0.032	0.136	0.024
AMHQ Total	**0.244**	**0.194**	**0.288**	0.108	**0.186**	**0.190**	0.128	0.071	0.019	0.076	-0.041

1.5. **Supplementary Table 5.** Pairwise correlations among items within the *Adult Math History Questionnaire*. (*Note*. Correlation coefficients in **bold** met $p < 0.05$.)

	Adult Math History Questionnaire											
**Item**	**1**	**2**	**3**	**4**	**5**	**6**	**7**	**8**	**9**	**10**	**11**	**12**
**Adult Math History Questionnaire**												
1	-											
2	**0.857**	-										
3	**0.727**	**0.661**	-									
4	**0.822**	**0.780**	**0.808**	-								
5	**0.663**	**0.567**	**0.651**	**0.706**	-							
6	**0.370**	**0.257**	**0.396**	**0.354**	**0.513**	-						
7	**0.453**	**0.378**	**0.414**	**0.459**	**0.624**	**0.501**	-					
8	**0.621**	**0.511**	**0.580**	**0.621**	**0.705**	**0.465**	**0.766**	-				
9	**0.550**	**0.517**	**0.474**	**0.612**	**0.549**	**0.288**	**0.509**	**0.651**	-			
10	**0.250**	**0.263**	**0.290**	**0.296**	**0.236**	**0.186**	**0.426**	**0.357**	**0.292**	-		
11	**0.155**	**0.150**	**0.198**	**0.228**	**0.227**	**0.189**	**0.277**	**0.330**	**0.348**	**0.281**	-	
12	**0.356**	**0.307**	**0.376**	**0.420**	**0.417**	**0.328**	**0.569**	**0.587**	**0.438**	**0.334**	**0.280**	-
13	**0.317**	**0.272**	**0.331**	**0.396**	**0.320**	**0.232**	**0.342**	**0.379**	**0.388**	**0.566**	**0.601**	**0.332**
14	**0.481**	**0.389**	**0.543**	**0.567**	**0.598**	**0.431**	**0.629**	**0.668**	**0.592**	**0.256**	**0.365**	**0.508**
15	**0.165**	0.129	**0.226**	**0.174**	**0.207**	**0.354**	**0.370**	**0.289**	0.127	**0.280**	**0.148**	**0.414**
16	**0.466**	**0.378**	**0.468**	**0.486**	**0.474**	**0.320**	**0.545**	**0.623**	**0.531**	**0.319**	**0.262**	**0.509**
17	**0.584**	**0.488**	**0.576**	**0.619**	**0.677**	**0.466**	**0.605**	**0.735**	**0.556**	**0.268**	**0.302**	**0.563**
AMHQ Total	**0.759**	**0.677**	**0.749**	**0.799**	**0.798**	**0.596**	**0.763**	**0.850**	**0.704**	**0.467**	**0.438**	**0.669**

	Adult Math History Questionnaire				
**Item**	**13**	**14**	**15**	**16**	**17**
**Adult Math History Questionnaire**					
13	-				
14	**0.333**	-			
15	**0.267**	**0.321**	-		
16	**0.401**	**0.554**	**0.336**	-	
17	**0.361**	**0.659**	**0.336**	**0.620**	-
AMHQ Total	**0.546**	**0.753**	**0.448**	**0.700**	**0.816**

1.6. Supplementary Table 6. Pairwise correlations between items from the *Adult Math History Questionnaire* (columns) and those from the *Adult Reading History Questionnaire* (rows). (*Note*. Correlation coefficients in **bold** met $p < 0.05$.)

	Adult Math History Questionnaire											
**Item**	**1**	**2**	**3**	**4**	**5**	**6**	**7**	**8**	**9**	**10**	**11**	**12**
**Adult Reading History Questionnaire**												
1	**0.287**	**0.303**	**0.337**	**0.358**	**0.215**	0.116	**0.141**	**0.168**	**0.165**	**0.166**	0.117	**0.181**
2	**0.380**	**0.381**	**0.375**	**0.400**	**0.279**	0.078	0.094	**0.227**	**0.269**	0.062	**0.138**	0.034
3	**0.314**	**0.328**	**0.387**	**0.390**	**0.275**	0.044	0.019	**0.166**	**0.274**	0.098	0.113	0.054
4	**0.138**	**0.220**	**0.167**	0.176	0.098	0.075	0.082	**0.150**	**0.197**	0.109	0.064	0.074
5	**0.210**	**0.281**	**0.186**	**0.222**	**0.172**	0.039	0.024	**0.145**	**0.206**	0.057	0.100	0.084
6	**0.267**	**0.273**	**0.426**	**0.348**	**0.210**	0.073	-0.010	**0.144**	**0.297**	0.110	**0.169**	0.079
7	**0.534**	**0.512**	**0.604**	**0.604**	**0.457**	**0.202**	**0.173**	**0.371**	**0.422**	**0.193**	**0.186**	**0.207**
8	**0.199**	**0.251**	**0.196**	0.182	0.096	-0.067	-0.065	0.027	**0.148**	0.032	-0.030	0.032
9	0.129	0.118	0.123	0.150	0.065	0.029	-0.019	0.064	0.120	0.023	0.067	0.061
10	0.015	-0.003	0.064	0.018	-0.009	0.038	0.007	-0.027	0.001	-0.030	-0.072	0.002
11	0.114	0.137	**0.248**	0.185	0.087	-0.018	-0.135	0.098	**0.173**	-0.126	0.093	-0.047
12	0.012	-0.020	0.006	0.021	-0.002	0.061	0.068	0.066	0.025	0.103	**0.219**	0.099
13	**0.328**	**0.367**	**0.364**	0.379	**0.221**	0.011	0.084	**0.177**	**0.204**	0.106	0.072	0.029
14	**0.225**	**0.249**	**0.287**	0.254	0.092	0.005	0.024	0.092	**0.155**	0.111	**0.145**	0.045
15	**0.326**	**0.358**	**0.316**	0.302	**0.243**	0.080	0.086	**0.188**	**0.277**	0.032	0.084	**0.201**
16	**0.154**	0.072	0.081	0.179	**0.146**	0.017	0.109	0.113	**0.161**	-0.013	-0.074	0.078
17	**0.230**	**0.182**	**0.143**	0.232	**0.219**	0.150	0.124	**0.166**	**0.269**	0.010	-0.029	0.048
18	**0.213**	**0.162**	0.111	0.246	**0.162**	0.040	0.068	**0.139**	**0.264**	0.000	0.085	0.092
19	**0.139**	**0.165**	**0.158**	0.165	0.080	0.036	0.004	0.127	**0.198**	0.003	0.034	0.057
20	0.096	**0.111**	**0.147**	0.123	0.061	0.048	-0.014	0.025	0.050	0.050	-0.037	-0.031
21	-0.035	-0.041	-0.042	-0.032	-0.013	0.065	-0.043	0.032	0.002	-0.021	-0.039	0.128
22	0.097	0.075	0.068	0.117	0.051	0.123	-0.025	0.019	0.058	-0.015	0.031	0.021
23	-0.086	-0.023	-0.086	-0.037	-0.090	-0.010	-0.052	-0.026	-0.020	-0.061	-0.023	0.057
ARHQ Total	**0.354**	**0.368**	**0.382**	**0.412**	**0.249**	0.094	0.063	**0.218**	**0.314**	0.077	0.104	0.129

	Adult Math History Questionnaire				
**Item**	**13**	**14**	**15**	**16**	**17**
**Adult Reading History Questionnaire**					
1	**0.234**	**0.188**	**0.157**	**0.268**	**0.250**
2	**0.148**	**0.254**	-0.008	**0.246**	**0.220**
3	**0.156**	**0.237**	0.008	**0.225**	**0.198**
4	0.091	0.084	0.066	**0.166**	0.044
5	**0.149**	0.048	-0.020	**0.139**	0.110
6	**0.212**	**0.229**	0.031	**0.226**	**0.204**
7	**0.287**	**0.367**	0.107	**0.406**	**0.400**
8	0.082	0.018	-0.028	**0.177**	0.115
9	0.114	0.018	0.062	0.092	0.096
10	-0.021	-0.051	0.058	0.036	-0.003
11	0.047	**0.147**	-0.005	0.117	**0.145**
12	**0.331**	0.046	**0.220**	0.079	0.068
13	0.131	**0.193**	-0.052	0.135	0.137
14	**0.145**	**0.211**	0.034	**0.167**	**0.177**
15	0.123	**0.208**	0.001	**0.290**	**0.217**
16	0.033	0.082	-0.050	0.123	0.091
17	0.036	0.111	-0.027	0.060	**0.162**
18	0.126	0.135	-0.063	**0.184**	**0.174**
19	-0.030	0.095	-0.018	**0.158**	0.055
20	0.004	-0.020	0.092	0.038	0.062
21	0.046	0.043	**0.157**	0.033	0.032
22	0.070	0.076	**0.141**	0.036	0.136
23	0.020	-0.024	0.043	0.019	0.024
ARHQ Total	**0.196**	**0.218**	0.077	**0.272**	**0.261**

1.7. Supplementary Table 7. Pairwise correlations between self-report (ARHQ and AMHQ; including total and factor scores [*italicized*] under each scale) and performance-based data collected from parents. (*Note*. Analyses were conducted for both scores on individual subtests and composite clusters from the *Woodcock-Johnson III* [Woodcock et al., 2001]. Correlation coefficients in **bold** met $p < 0.05$.)

		1	2	3	4	5	6	7	8	9	10
**Self-Report Measures**											
Reading											
1	ARHQ (Total)	-									
2	*Childhood Ability*	**0.80**	-								
3	*Attitude & Exposure*	**0.55**	**0.27**	-							
4	*Memory*	**0.60**	**0.26**	**0.22**	-						
5	*Media Use*	**0.40**	0.01	**0.28**	**0.28**	-					
6	*Reversal*	**0.44**	**0.39**	0.08	**0.22**	-0.06	-				
7	*Spelling*	**0.68**	**0.64**	**0.19**	**0.30**	0.03	**0.34**	-			
Math											
8	AMHQ (Total)	**0.33**	**0.37**	0.05	**0.19**	-0.01	**0.18**	**0.24**	-		
9	*Childhood Ability*	**0.41**	**0.48**	0.07	**0.24**	-0.03	**0.19**	**0.34**	**0.81**	-	
10	*Attitude & Exposure*	**0.24**	**0.27**	0.02	**0.18**	0.00	0.13	**0.17**	**0.95**	**0.68**	-
**Performance-Based Measures**											
Reading											
11	Letter-Word Identification	**-0.43**	**-0.47**	**-0.23**	0.00	-0.12	-0.13	**-0.48**	**-0.17**	**-0.21**	-0.12
12	Word Attack	**-0.31**	**-0.34**	-0.15	0.03	0.00	**-0.16**	**-0.40**	-0.15	**-0.21**	-0.10
13	Sentence Reading Fluency	**-0.42**	**-0.45**	**-0.24**	0.04	-0.11	**-0.18**	**-0.37**	-0.02	-0.09	0.03
14	Basic Reading (Composite)	**-0.41**	**-0.45**	**-0.21**	0.01	-0.07	-0.15	**-0.48**	**-0.17**	**-0.23**	-0.12
Math											
15	Calculation	**-0.20**	**-0.31**	0.00	0.08	0.00	**-0.21**	**-0.23**	**-0.53**	**-0.51**	**-0.49**
16	Math Facts Fluency	**-0.30**	**-0.30**	-0.03	-0.02	-0.11	**-0.20**	**-0.31**	**-0.41**	**-0.43**	**-0.38**
17	Math Calculation (Composite)	**-0.28**	**-0.34**	-0.02	0.00	-0.06	**-0.22**	**-0.30**	**-0.52**	**-0.52**	**-0.48**
**Background Measure**											
18	Educational Attainment	**-0.23**	**-0.26**	**-0.18**	0.00	**-0.17**	-0.06	-0.10	**-0.21**	**-0.16**	**-0.21**

		11	12	13	14	15	16	17	18
**Performance-Based Measures**									
Reading									
11	Letter-Word Identification	-							
12	Word Attack	**0.66**	-						
13	Sentence Reading Fluency	**0.60**	**0.45**	-					
14	Basic Reading (Composite)	**0.93**	**0.89**	**0.58**	-				
Math									
15	Calculation	**0.44**	**0.36**	**0.33**	**0.44**	-			
16	Math Facts Fluency	**0.35**	**0.30**	**0.53**	**0.36**	**0.64**	-		
17	Math Calculation (Composite)	**0.44**	**0.37**	**0.48**	**0.44**	**0.90**	**0.92**	-	
**Background Measure**									
18	Educational Attainment	**0.34**	**0.22**	**0.31**	**0.31**	**0.35**	**0.28**	**0.35**	-

1.8. Supplementary Table 8. Pairwise correlations between scores (total and factor) on the ARHQ and AMHQ and measures of children's core academic skills (word recognition [WR] and arithmetic calculation [AR]), and performance on complex tasks (reading comprehension [RC] and problem solving [PS]). (*Note*. Correlation coefficients in **bold** met $p < 0.05$.)

	**ARHQ**							**AMHQ**		
	**Total**	Childhood Ability	Attitude & Exposure	Memory	Media Use	Reversal	Spelling	**Total**	Childhood Ability	Attitude & Exposure
**Core Academic Skills**										
WR (after 1st grade)	**-0.19**	**-0.24**	-0.11	0.08	-0.07	-0.07	**-0.16**	-0.08	**-0.20**	-0.06
WR (after 2nd grade)	**-0.17**	**-0.20**	-0.10	0.07	-0.09	-0.06	-0.11	-0.04	-0.15	-0.04
AR (after 1st grade)	**-0.14**	**-0.15**	-0.09	0.04	-0.10	-0.03	-0.09	**-0.17**	**-0.23**	-0.12
AR (after 2nd grade)	**-0.20**	**-0.15**	-0.14	-0.06	**-0.19**	-0.13	-0.05	**-0.29**	**-0.31**	**-0.27**
**Complex Academic Tasks**										
RC (after 2nd grade)	**-0.18**	**-0.16**	**-0.16**	0.04	**-0.22**	0.04	0.00	0.00	-0.09	0.03
RC (after 3rd grade)	**-0.17**	-0.14	**-0.24**	0.06	-0.13	-0.04	0.06	-0.06	-0.10	-0.06
PS (after 2nd grade)	**-0.26**	**-0.26**	**-0.27**	0.01	**-0.19**	-0.09	-0.03	-0.13	**-0.21**	-0.09
PS (after 3rd grade)	-0.13	0.00	**-0.25**	-0.10	-0.16	0.02	0.09	0.00	0.02	0.00

1.9. Supplementary Table 9. Covariances among factors derived from the ARHQ and AMHQ reported in the final path model and **Supplementary Figure 2**. (*Note*. All coefficients shown met $p < 0.05$.)

		1	2	3	4	5	6	7	8
	**ARHQ Factors**								
1	Childhood Ability	-							
2	Attitude & Exposure	0.206	-						
3	Memory	0.137	0.200	-					
4	Media Use	-	0.266	0.278	-				
5	Reversal	0.304	-	-	-	-			
6	Spelling	0.576	0.136	0.206	-	0.251	-		
	**AMHQ Factors**								
7	Childhood Ability	0.271	-	0.205	-	-	0.194	-	
8	Attitude & Exposure	-	-	1.450	-	-	-	0.600	-

2. Supplementary Figure

2.1. Supplementary Figure 1. Initial (full) path model to be evaluated for the direct and indirect effects of familial history of reading and math difficulties (i.e., indexed by ARHQ and AMHQ, respectively) on academic measures on children's core academic skills (word reading [WR] and arithmetic calculation [AR]) after first and second grades, and performance on complex tasks (reading comprehension [RC] and problem solving [PS]) after second and third grades.

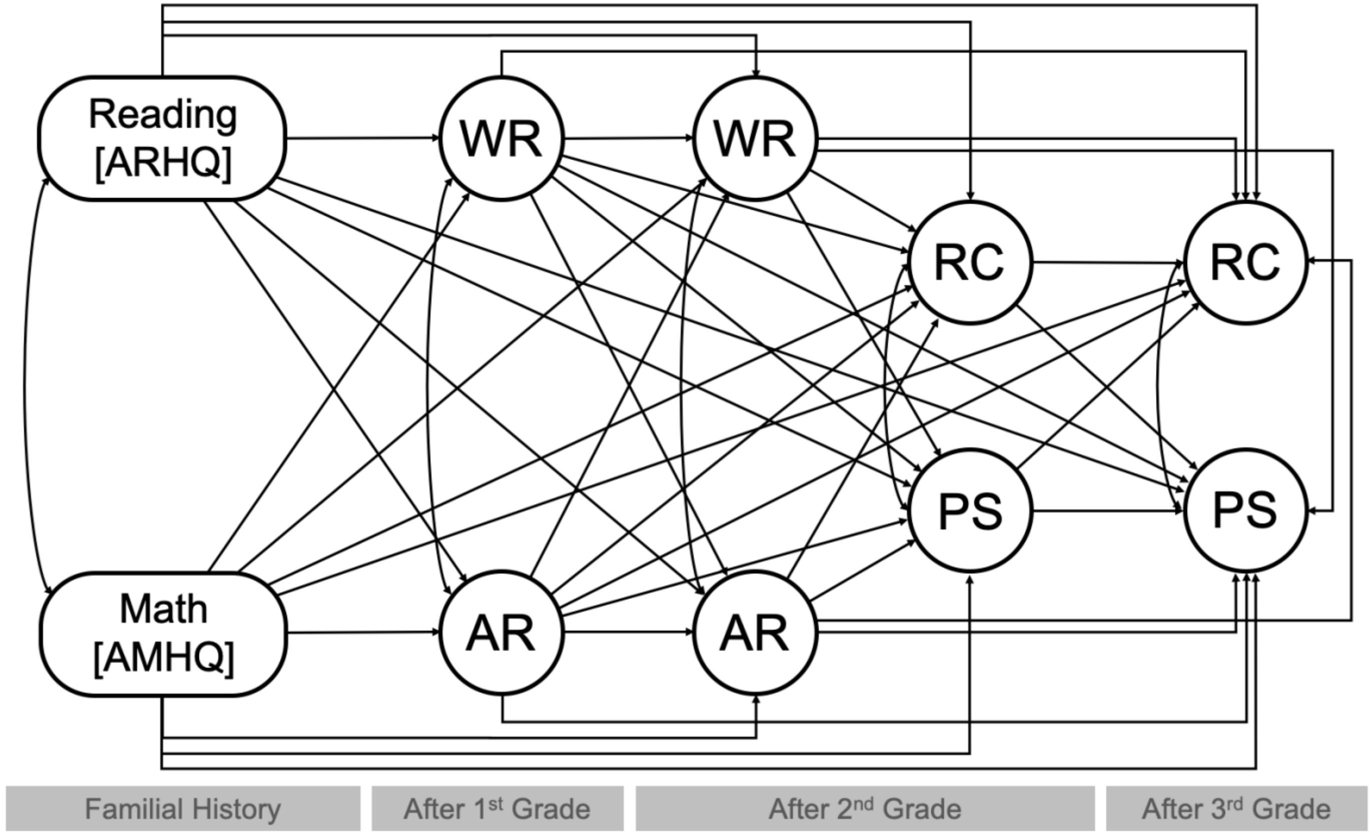

3. Supplementary Materials and Methods

To address the gap in the literature such that there is currently no validated scale that captures familial history of math difficulties that mirror the *Adult Reading History Questionnaire* (ARHQ; Lefly & Pennington, 2000) (or shown to be correlated with current parental academic functioning), the *Adult Math History Questionnaire* (AMHQ) was designed and implemented when the current longitudinal study began in 2015 in order to track the role of familial math history in children's academic outcomes over time. Specifically, *Items 1*, *2*, *3*, and *4* survey the respondents' experiences with math learning and related contents in elementary school, whereas *Items 5* and *6* with materials in upper education (high school and college). *Items 7, 8*, and *10* target the respondents' (current) attitude toward math and related contents, while *Items 14, 15, 16*, and *17* also place an emphasis on confidence and interest in math. *Items 9*, *11*, *12*, and *13* inquire about the respondents' current numeracy practices and math exposure.

4. Supplementary Results

4.1. Psychometric Findings

Item-level loading coefficients for individual factors derived from the ARHQ and AMHQ can be found in **Tables 3** and **4**.

- <u>*Adult Reading History Questionnaire*</u> (ARHQ):
  - [Factor 1] Childhood Ability includes [*Item 3*] "How much extra help did you need when learning to read in elementary school?" (loading coef. = 0.832), [*Item 2*] "How much did difficulty did you have learning to read in elementary school?" (loading coef. = 0.822), [*Item 15*] "Did your parents ever consider having you repeat any grades in school due to academic failure (not illness)?" (loading coef. = 0.718), [*Item 6*] "How would you compare your reading skill to that of others in your elementary classes?" (loading coef. = 0.697), and [*Item 7*] "All students struggle from time to time in school. In comparison to others in your classes, how much did you struggle to complete your work?" (loading coef. = 0.638). Overall, these items focused on how parents functioned in school and elementary education, thus reflecting Childhood Ability in reading.
  - [Factor 2] Attitude/Exposure includes [*Item 10*] "How much reading do you do for pleasure? (loading coef. = 0.952) and [*Item 20*] "How many books do you read for pleasure each year?" (loading coef. = 0.849). These items highlighted parents' current reading and literacy practices, thus indicating Attitude/Exposure in reading.
  - [Factor 3] Memory includes [*Item 16*] "Do you ever have difficulty remembering people's names or names of places?" (loading coef. = 0.901), [*Item 17*] "Do you every have difficulty remembering addresses, phone numbers, or dates?" (loading coef. = 0.717), and [*Item 18*] "Do you have difficulty remembering complex verbal instructions?" (loading coef. = 0.649). These items queried parents' memory abilities across different contexts (in terms of, e.g., names, numbers, dates, and instructions), thus illustrating Memory.
  - [Factor 4] Media Use includes [*Item 23*] "Do you read a newspaper on Sunday?" (loading coef. = 0.918) and [*Item 22*] "Do you read daily (Monday-Friday) newspapers?" (loading coef. = 0.725). These items inquired about parents' current usage of print and media, particularly newspapers, thus revealing Media Use.
  - [Factor 5] Reversal includes [*Item 4*] "Did you ever reverse the order of letters or numbers when you were a child?" (loading coef. = 0.995). This item asked about parents' previous experience with switching the order of letters or numbers as a child, thus representing Reversal.
  - [Factor 6] Spelling includes [*Item 14*] "How would you compare your current spelling to that of others of the same age and education?" (loading coef. = 0.913). This item questioned about parents' current spelling ability relative to their peers (of same age and education), thus implicating Spelling.
- <u>*Adult Math History Questionnaire*</u> (AMHQ):
  - [Factor 1] Attitude/Exposure includes [*Item 7*] "What is your current attitude towards math?" (loading coef. = 0.896), [*Item 8*] "Math makes me feel uncomfortable and nervous." (loading coef. = 0.814), [*Item 14*] "How would you compare your current math skills to those of others of the same age and education?" (loading coef. = 0.755), [*Item 12*] "I enjoy completing math and logic puzzles for fun." (loading coef. = 0.736), [*Item 17*] "New math content has usually been easy and enjoyable for me to understand."

(loading coef. = 0.709), and [*Item 16*] "I feel confident in helping my child with their math and homework (loading coef. = 0.667). *Items 7, 8, 12, 14,* and *17* though with different phrasing (i.e., in forms of a question or statement) showed high correlations (r's = 0.508 – 0.766, $p < 0.05$; *mean* $r = 0.626$) and, as such, loaded highly (coefficients > 0.700) into one construct characterizing parents' current attitude towards math and numeracy practices – or Attitude/Exposure in math.
    - [Factor 2] Childhood Ability includes [*Item 2*] "When in elementary school, I needed extra help in math from a teacher or tutor." (loading coef. = 0.965), [*Item 1*] "When in elementary school, I struggled with learning new concepts in math." (loading coef. = 0.914), [*Item 4*] "Compared to others in your elementary classes, how much did you struggle to complete your math work?" (loading coef. = 0.803), and [*Item 3*] "How would you compare your math skills to those of others in your elementary classes?" (loading coef. = 0.678). *Items 1, 2,* and *4* though with different phrasing showed high correlations (r's = 0.780 – 0.857, $p < 0.05$; *mean* $r = 0.819$) and, as such, loaded highly (coefficients > 0.700) into one construct describing parents' self-reported experiences with learning math and related contents in elementary school – or Childhood Ability in math.

Pairwise correlations between factor scores on the ARHQ and AMHQ and performance-based measures of parents' academic skills can be found in **Supplementary Table 7**.

- *Adult Reading History Questionnaire* (ARHQ):
    - The Childhood Ability factor was found to be significantly and positively correlated with the Attitude/Exposure, Memory, Reversal, and Spelling factors (r = 0.26 – 0.64, all p < 0.05), but not with the Media Use factor ($p > 0.05$). The Attitude/Exposure factor was significantly and positively correlated with the Memory, Media Use, and Spelling factors (r = 0.19 – 0.28, all $p < 0.05$), but not with the Reversal factor ($p > 0.05$). The Memory factor was significantly and positively correlated with the Media Use, Reversal, and Spelling factors (r = 0.22 – 0.30, all $p < 0.05$). The Media Use factor was not correlated with either the Reversal or Spelling factors ($p < 0.05$). The Reversal factor was significantly and positively correlated with the Spelling factor ($r = 0.34$, $p < 0.05$).
    - The Childhood Ability factor was found to be significantly and negatively correlated with reading scores (r = -0.34 – -0.47), as well as with math scores (r = -0.30 – -0.34) (all p < 0.05). The Attitude/Exposure factor was significantly and negatively correlated with reading scores (r = -0.21 – -0.24, all $p < 0.05$; with the exception of the Word Attack subtest, $r = -0.15$, $p > 0.05$) but not with any of the math scores ($p > 0.05$). The Memory factor was not correlated with any of the reading or math scores ($p > 0.05$). The Media Use factor was not correlated with any of the reading or math scores ($p > 0.05$). The Reversal factor was significantly and negatively correlated with scores on the Word Attack and Sentence Reading Fluency subtests for reading (r = -0.16 and -0.18, respectively), as well as with math scores (r = -0.20 – -0.22) (all $p < 0.05$). The Spelling factor was significantly and negatively correlated with reading scores (r = -0.37 – -0.48), as well as with math scores (r = -0.23 – -0.31) (all $p < 0.05$).
- *Adult Math History Questionnaire* (AMHQ):
    - The Childhood Ability factor was found to be significantly and positively correlated with the Attitude/Exposure factor ($r = 0.68$, $p < 0.05$).

  - The Childhood Ability factor in the ARHQ was found to be significantly and positively correlated with the Childhood Ability and Attitude/Exposure factors in the AMHQ (r = 0.48 and 0.27, respectively, both $p < 0.05$). The Attitude/Exposure factor in the ARHQ was not correlated with any of the factors in the AMHQ ($p > 0.05$). The Memory factor in the ARHQ was significantly and positively correlated with the Childhood Ability and Attitude/Exposure factors in the AMHQ (r = 0.24 and 0.18, both $p < 0.05$). The Media Use factor in the ARHQ was not correlated with any of the factors in the AMHQ ($p > 0.05$). The Reversal factor in the ARHQ was significantly and positively correlated with the Childhood Ability factor in the AMHQ ($r = 0.19$, $p < 0.05$). The Spelling factor in the ARHQ was significantly and positively correlated with the Childhood Ability and Attitude/Exposure factors in the AMHQ (r = 0.34 and 0.17, both $p < 0.05$).
  - The Childhood Ability factor was found to be significantly and negatively correlated with math scores (r = -0.43 – -0.53, all $p < 0.05$), as well as with reading scores (r = -0.21 – -0.23, all $p < 0.05$; with exception of the Sentence Reading Fluency subtest, $r = -0.09$, $p > 0.05$). The Attitude/Exposure factor was significantly and negatively correlated with math scores (r = -0.38 – -0.49, all $p < 0.05$), but not with any of the reading scores ($p > 0.05$).

4.2. Correlational Findings

Pairwise correlations between scores on individual factors derived from the ARHQ and AMHQ and measures of children's academic performance can be found in **Supplementary Table 8**. Note that the directionality for the computed factor scores remain consistent with the directionality of the questions on each scale – that is, higher scores on any factors extracted from the ARHQ or AMHQ indicate, e.g., increased difficulties with learning to read or do simple arithmetic (see **Materials and Methods**).

- *Adult Reading History Questionnaire* (ARHQ):
  - The Childhood Ability factor was found to be significantly and negatively correlated with children's WR skills measured after first ($r = -0.24$) and second grades ($r = -0.20$), as well as with AR abilities captured after first ($r = -0.15$) and second grades ($r = -0.15$) (all $p < 0.05$). The Childhood Ability factor was also significantly and negatively correlated with children's performance on RC task administered after second grade ($r = -0.16$), as well as on PS assessment collected after second grade ($r = -0.26$) (both $p < 0.05$).
  - The Attitude/Exposure factor was significantly and negatively correlated with children's performance on RC tasks administered after second ($r = -0.16$) and third grades ($r = -0.24$), as well as on PS assessments collected after second ($r = -0.27$) and third grades ($r = -0.25$) (all $p < 0.05$).
  - The Media Use factor was significantly and negatively correlated with children's AR ability measured after second grade ($r = -0.19$), as well as performance on RC task ($r = -0.22$) and PS assessment ($r = -0.19$) collected after second grade (all $p < 0.05$).
  - The Spelling factor was significantly and negatively correlated with children's WR skill measured after first grade ($r = -0.16$, $p < 0.05$).
- *Adult Math History Questionnaire* (AMHQ):
  - The Childhood Ability factor was found to be significantly and negatively correlated with children's WR skill measured after first grade ($r = -0.20$), as well as AR abilities captured after first ($r = -0.23$) and second grades ($r = -0.31$) (all $p < 0.05$). The Childhood Ability

factor was also significantly and negatively correlated with children's performance on PS assessment collected after second grade ($r = -0.21$, $p < 0.05$).
    - The Attitude/Exposure factor was significantly and negatively correlated with children's AR ability captured after second grade ($r = -0.27$, $p < 0.05$).

4.3. Path Findings

The initial model was reported with $\chi^2 = 48.923$ ($p = 0.002$), *CFI* = 0.980, *TLI* = 0.832, *RMSEA* = 0.087 ($p = 0.044$), and *SRMR* = 0.058, indicating a fair fit. To improve model fit, non-significant paths were constrained to zero. The final model was reported with $\chi^2 = 173.946$ ($p = 0.051$), *CFI* = 0.977, *TLI* = 0.968, *RMSEA* = 0.038 ($p = 0.825$), and *SRMR* = 0.041, indicating a good fit. This step in constraining non-significant paths to zero did not significantly improve the fit ($\Delta\chi^2 = 125.02$, $p = 0.383$) when comparing the initial (full) and final models, though the latter was more parsimonious and thus subjected to further steps to evaluate the direct and indirect effects of individual factors derived from the ARHQ and AMHQ (see **Figure 2**). Summary findings from follow-up analyses to distinguish the effects of individual factors derived from the ARHQ and AMHQ on measures of children's academic performance in **Table 7** and are discussed below. Note that the directionality for the computed factor scores remain consistent with the directionality of the questions on each scale – that is, higher scores on any factors extracted from the ARHQ or AMHQ indicate, e.g., increased difficulties with learning to read or do simple arithmetic (see **Materials and Methods**).

4.3.1. Direct and Indirect Effects on Core Academic Skills

- <u>*Familial history of reading difficulties*</u> (indexed by sub-scores on the ARHQ):
    - The Childhood Ability factor was shown to have a direct and negative effect on children's WR skill captured after first grade ($b = -0.177$), which in turn had an indirect effect on WR outcome assessed after second grade ($b = -0.158$) ($p < 0.05$). The Childhood Ability factor also appeared to have an indirect on children's AR outcome evaluated after second grade via WR skill measured after first grade ($b = -0.026$, $p < 0.05$).
- <u>*Familial history of math difficulties*</u> (indexed by sub-scores on the AMHQ):
    - The Childhood Ability factor was revealed to have a direct and negative effect on children's AR ability captured after first grade ($b = -0.171$), which in turn had an indirect effect on AR outcome assessed after second grade ($b = -0.112$) ($p < 0.05$).
    - The Attitude/Exposure factor was demonstrated to have a direct and negative effect on children's AR outcome evaluated after second grade ($b = -0.214$, $p < 0.05$).

4.3.2. Direct and Indirect Effects on Performance on Complex Academic Tasks

- <u>*Familial history of reading difficulties*</u>:
    - The Childhood Ability factor was shown to have an indirect effect on children's performance on RC task evaluated after second grade via the serial effect of WR skills measured after first and second grades ($b = -0.089$, $p < 0.05$). Such indirect effect of the Childhood Ability factor on RC performance captured after second grade in turn had an

impact on children's RC outcome assessed after third grade (b = -0.053, $p < 0.05$). The Childhood Ability factor also appeared to have an indirect on children's performance on PS task measured after second grade via the serial effects of WR skill captured after first grade on AR ability after second grade (b = -0.010, $p < 0.05$). Such indirect effect of familial history on PS performance assessed after second grade thereby had an impact on children's PS outcome evaluated after third grade (b = -0.002, $p < 0.05$).

  - The Media Use factor was revealed to have a direct and negative effect on children's performance on RC task measured after second grade (b = -0.131), in turn had indirect effects on their RC outcome determined after third grade (b = -0.078) ($p < 0.05$). The Media Use factor also appeared to have an indirect effect on children's performance on PS assessment after third grade via RC outcome captured after third grade (b = -0.010, $p < 0.05$).
  - The Attitude/Exposure factor was demonstrated to have direct effects on children's RC outcome evaluated after third grade (b = -0.107), as well as on their performance on PS task assessed after second grade (b = -0.109) ($p < 0.05$). Such direct effect from the Attitude and Exposure factor on children's PS performance after second grade in turn had an impact on their PS outcome measured after third grade (b = -0.027, $p < 0.05$).
- *Familial history of math difficulties*:
  - The Childhood Ability factor was reported to have an indirect effect on children's performance on PS task evaluated after second grade via the serial effects of AR skills measured after first and second grades (b = -0.043, $p < 0.05$). Such indirect effect of the Childhood Ability factor on PS performance captured after second grade in turn had an impact on children's PS outcome assessed after third grade (b = -0.011, $p < 0.05$).
  - The Attitude/Exposure factor was shown to have an indirect effect on children's PS outcomes evaluated after second via their AR skill assessed after second grade (b = -0.081; $p < 0.05$). The Attitude/Exposure factor appeared to also have an indirect on children's PS outcome captured after third grade via the concurrent relation between the measures of their AR and PS abilities evaluated after second grade (b = -0.020, $p < 0.05$).
